# Top-down enrichment of oil-degrading microbial consortia reveals functional streamlining and novel degraders

**DOI:** 10.3389/fmicb.2025.1656448

**Published:** 2025-12-03

**Authors:** Huijun Wu, Xianyuan Du, Jin Zheng, Xingchun Li, Quanwei Song, Yuhao Yan, Anzhou Ma, Anming Xu, Jufeng Li

**Affiliations:** 1 State Key Laboratory of Petroleum Pollution Control, China National Petroleum Corporation Research Institute of Safety and Environment Technology, Beijing, China; 2 College of Materials Science and Engineering, China University of Geosciences, Beijing, China; 3 University of the Chinese Academy of Sciences, Beijing, China; 4 College of Biotechnology and Pharmaceutical Engineering, Nanjing Tech University, Nanjing, China

**Keywords:** top-down enrichment strategy, microbial consortia, petroleum hydrocarbon, bioremediation, correlation network

## Abstract

**Introduction:**

Top-down microbial enrichment is a reliable approach for understanding and designing microbiomes for crude oil remediation. Environmental variables serve as valuable determinants for selecting desired microbiomes with superior performance. However, the linkages between selection methods and the structure and function of desired microbiomes remain unclear.

**Methods:**

This study integrated substrate concentration gradients and cultivation patterns to investigate how selection pressures shape top-down enriched crude oil-degrading consortia. The resulting communities were analyzed using 16S rRNA gene sequencing, metagenomics, and co-occurrence network analysis. Key bacterial strains were isolated to validate their individual degradation capabilities.

**Results:**

The top-down process led to a significant reduction in phylogenetic diversity but a notable increase in the potential for xenobiotic degradation and metabolic. The final consortia, GT4, achieved a 55.72% degradation rate of crude oil at an initial concentration of 5 g/L within 7 days. Metagenomic analysis identified *Microbacterium* as dominant genus harboring key enzymes for the degradation of alkanes and aromatic compounds. Co-occurrence network analysis revealed *Mesorhizobium* as a keystone genus, showing positive associations with multiple diazotrophic bacteria and hydrocarbon degrading bacteria. Nine bacterial strains were isolated from the consortium. Among them, *Microbacterium* sp. WS3 and *Cellulosimicrobium* sp. WS9 exhibited high degradation efficiencies (57.85 and 58.60%, respectively). To the best of our knowledge, this study provides the first experimental evidence for crude oil degradation by *Paracandidimonas* and *Caulobacter*, with degradation rates of 51.19 and 40.90%, respectively.

**Discussion:**

These findings highlight the effectiveness of top-down enrichment strategy in generating functionally streamlined consortia and uncover novel oil-degrading microbes with potential for bioremediation applications.

## 1 Introduction

Bioremediation utilizes microorganisms to degrade organic and inorganic pollutants in contaminated environments. Compared to single strains, microbial consortia often demonstrate superior efficiency by integrating diverse metabolic pathways from multiple species, thereby enhancing environmental tolerance and broaden degradation capacity (Cui et al., 2020; Gurav et al., 2017; Mukherjee and Bordoloi, 2012; Zhang and Zhang, 2022). Methods for constructing microbial consortia can be categorized into bottom-up and top-down approaches (San León and Nogales, 2022). The bottom-up approach involves assembling individual strains to construct more complex microbial systems. Such combinations are typically guided by the functional strengths or complementarity of the selected strains, although the process is often empirical. For example, oil-degrading and 2,4-dichlorophenol-degrading bacteria have been co-cultured to form a highly efficient consortium capable of degrading crude oil in the presence of chlorophenol compounds (Li J. et al., 2022). In contrast, the top-down approach seeks to reduce community complexity by applying selective enrichment strategies (Tshikantwa et al., 2018). This method has been employed to enrich microbial consortia with hydrocarbon-degrading capabilities. For instance, a consortium dominated by *Marinobacter* and *Alcanivorax* was shown to grow using 13 g/L of diesel as the sole carbon source (Garcia-Cruz et al., 2019). Blanco-Enríquez et al. enriched a consortium dominated by *Stenotrophomonas, Williamsia*, and Chitinophagaceae, which removed 90% of the PAHs at an initial concentration of 100 mg/L within 14 days (Blanco-Enriquez et al., 2018). Similarly, consortium EC20 enriched from Bohai Sea sediment, was dominated by *Pseudomonas, Mesorhizobium, Achromobacter, Stenotrophomonas,* and *Halomonas,* and demonstrated 87% degradation of BTEX compounds at an initial concentration of 435 mg/L (Deng et al., 2017).

The community structure of top-down consortia can vary significantly depending on the sample source, cultivation conditions, and target pollutants (Viñas et al., 2002; Xiong et al., 2015). When applied to mixed pollutants, both the taxonomic and metabolic profiles of these consortia tend to fluctuate (Chen et al., 2019; Lewin et al., 2022). Owing to the complex composition of crude oil, the structure and function of oil-degrading consortia are subject to constant dynamic change. However, few studies have systematically investigated the evolution dynamics of these communities during the enrichment process. It is therefore critical to develop enrichment strategies that promote both enhanced degradation function and structural stability. Top-down enrichment may help preserve intrinsic microbial interactions within consortia (Borchert et al., 2021). Advances in high-throughput sequencing have facilitated co-occurrence network analysis, enabling the identification of potential syntrophic relationships among microbial taxa (Dai et al., 2022; Li et al., 2023; Ya et al., 2022). In parallel, meta-analyses have been used to predict key functional enzymes and microbial taxa involved in hydrocarbon degradation (Wongbunmak et al., 2020; Wu et al., 2023). Together, amplicon sequencing and meta-omics provide powerful tools for dissecting the taxonomic composition and metabolic potential of crude oil-degrading consortia.

In this study, a top-down strategy was evaluated under varying cultivation durations and crude oil concentrations to guide microbial community evolution. The microbial consortia were enriched from the crude oil-contaminated soil collected in the oilfield from Karamay (Xinjiang China). This oilfield is one of the oldest and largest in China, with a long history of petroleum extraction and processing spanning several decades. Crude oil used as substrate in the enrichment process was also collected from the Xinjiang oil field. The composition of this crude oil, as determined in a previous study (Chunyang et al., 2019), was 71.1% saturated hydrocarbons, 10.6% aromatic hydrocarbons, 12.1% resin, and 6.2% asphaltene. High-throughput sequencing was employed to track microbial community dynamics across different transfer generations and cultivation periods. Unlike the bottom-up strategies that rely on predefined organisms or metabolic pathways, the top-down approach utilizes selected environmental variables to impose ecological pressures on an existing microbiome, thereby steering the consortia toward the desired biological function (Lawson et al., 2019). This approach facilitates the development of degradative microbial consortia harboring intricate metabolic interactions and enhanced functional synergy (Gilmore et al., 2019). The results of this study indicated that the combination of a relatively high substrate concentration and prolonged cultivation led to the formation of high-performance consortia with reduced complexity. Within the consortia, diazotrophic bacteria were identified to play a syntrophic role, highlighting their potential utility in the rational design of co-cultures for crude oil degradation. Overall, this study presents an effective approach for developing top-down microbial consortia and provides new insights into the ecological interactions driving crude oil biodegradation.

## 2 Materials and methods

### 2.1 Soil sampling and physicochemical characterization

Contaminated soil samples and crude oil were obtained from the Karamay oilfield, located on the northwestern edge of the Junggar Basin in Xinjiang, Northwest China. This area lies within the arid region of China and is characterized predominantly by flat Gobi desert terrain, with an average elevation of approximately 400 meters. The climate is typically temperate continental, with an average annual precipitation of less than 110 mm. The forest coverage rate of the region is approximately 16.4%. Five surface soil samples (0–20 cm depth) were collected within the contaminated area using a five-point sampling method. Samples were sealed in sterilized polyethylene bags, then delivered to the laboratory on ice. These five sub-samples were homogenized to form one composite sample and sieved through a 2 mm mesh.

Soil pH and salinity were determined at a soil-to-water ratio of 1:2.5 (w/v) and 1:5 (w/v), respectively, using a multiparameter meter. Soil moisture content was determined gravimetrically by measuring the mass loss after oven-drying at 105 °C to constant weight (approximately 24 h). Organic matter content was determined using the Walkley-Black method, which involves wet oxidation with potassium dichromate in sulfuric acid. Total nitrogen (TN) content was measured using a TOC analyzer (Multi N/C 3100 TOC, Analytik, Jena, Germany). Total petroleum hydrocarbon (TPH) content was quantified by extracting freeze-dried soil with a dichloromethane and acetone mixture, followed by purification and measurement. The characterized physicochemical properties of the polluted soil were as follows: pH 7.8, moisture content 9.0%, TPH 29,900 mg/kg, salinity 15.4 mg/kg, organic matter 90.4 g/kg, and total nitrogen content 0.8 g/kg.

### 2.2 Bacterial consortia enrichment strategy

The mineral salt medium (MM, pH ~ 7.5) supplemented with crude oil as sole carbon source was used for enrichment cultivation, microbial isolation, and biodegradation test in this study. The composition of the MM medium was (g/L): 1 K_2_HPO_4_, 1 KH_2_PO_4_, 1 NH_4_Cl, 2.24 MgSO_4_·7H_2_O, 0.05 FeCl_3_·6H_2_O, 0.02 CaCl_2_. Luria-bertani (LB) medium, composed of (g/L) 10 treptone, 5 NaCl and 5 yeast extract, was used for strain propagation. Crude oil was sterilized by UV treatment prior to use.

The enrichment cultures were divided into two regimes: serial subculturing and prolonged cultivation (Supplementary Figure S1). To detach microorganisms from soil particles, the soil suspension was prepared by adding 5 g of soil sample in a 150 mL Erlenmeyer flask containing 45 mL MM medium. The flasks were incubated on a horizontal shaker at 30 °C and 150 rpm for 30 min, and the resulting suspension was used as the seed liquid for the top-down enrichment process. Specifically, the suspension was transferred (10% v/v) into four 250 mL Erlenmeyer flasks, each containing 100 mL of MM medium with 5 g/L crude oil as sole carbon source. These four replicate flasks constituted Group 1 (G1). Subsequently, serial subculturing was performed every 30 days by transferring a 10% (v/v) inoculum from the previous group into fresh medium to generate the next group. Thus, after four subculturing cycles, a total of four transfer generations (G1 to G4) were obtained. All cultures were incubated in a horizontal shaker at 150 rpm and 30 °C, and cells were harvested at their respective 30-day time points. The crude oil concentration was increased from 5 g/L to 10 g/L at the fourth subculture (G4) to evaluate the impact of increased environmental pressure on the consortia.

To investigate the community response to extended stress, consortia from different substrate concentration (G3 and G4) and with distinct microbial diversity (G4) were selected for prolonged cultivation. During the prolonged cultivation phase, the incubation period for G3 and G4 was extended from 30 days to 90 days. The bacterial cultures harvested at 90 days were designated as prolonged cultures and named GT3 and GT4, respectively. Overall, the six groups (G1-G4, GT3, and GT4), each with four biological replicates, yielded a total of 24 culture samples for subsequent sequencing analysis.

### 2.3 16s rRNA gene sequencing and analysis

During the enrichment process, 1 mL culture was collected from each flask. The samples were centrifuged at 12000 rpm for 5 min, and the pellet was collected as the bacterial consortium. A total of 24 bacterial enrichment cultures (six groups with four biological replicates each) were subjected to microbial community analysis. Total genomic DNA of the bacterial enrichment cultures was extracted with the E. Z. N. A.® Soil DNA Kit (Omega Bio-tek, Norcross, GA, United States) using the manufacture’s protocol. The quality of DNA extracts were checked on 1% agarose gel. The V3–V4 region of 16S rRNA genes were amplified with the primer 338F (5′-ACTCCTACGGGAGGCAGCAG-3′) and 806R (5′-GGACTACHVGGGTWTCTAAT-3′) (Ma et al., 2015). Purified amplicons were pooled in equimolar amounts and subjected to paired-end sequencing on an Illumina MiSeq PE300 platform (Illumina, San Diego, United States) according to the standard protocols by Majorbio Bio-Pharm Technology Co. Ltd. (Shanghai, China) (Wu et al., 2023).

Raw FASTQ files were demultiplexed using an in-house Perl script, and then quality-filtered by fastp (version 0.19.6) (Chen et al., 2018) and merged by FLASH version 1.2.7 (Magoč and Salzberg, 2011). The processed sequences were clustered into operational taxonomic units (OTUs) at a 97% similarity threshold using UPARSE (version 11.0.667) (Edgar, 2013; STACKEBRANDT & GOEBEL Stackebrandt and Goebel, 1994). Finally, all samples were rarefied to an even sequencing depth of 25,617 per sample to ensure comparability for subsequent alpha- and beta-diversity analyses. Taxonomy assignment of each OTU representative sequence was performed using RDP Classifier (version 11.5) (Wang et al., 2007) against the SILVA 16S rRNA gene database (release v138.2) with a confidence threshold of 0.7.

Four alpha-diversity indices—the Shannon index, Inverse Simpson index, Chao1 estimator, and Phylogenetic diversity (PD)—were calculated based on the OTUs using Mothur (v1.30). Significance of differences the diversity indices was calculated by paired Student’s *t*-test and one-way ANOVA. Shifts in community structure were studied by non-metric multidimensional scaling (NMDS) based on Bray-Curtis distance, implemented in the vegan package (v2.5–3). Predictive function profiling of microbial communities was performed using PICRUSt2 (phylogenetic investigation of communities by reconstruction of unobserved states) based on KEGG orthologs and pathway level 3 annotations (Langille et al., 2013). A low Nearest Sequenced Taxon Index (NSTI) value (0.18 ± 0.24) indicated a high accuracy of the metagenome prediction. Significance of differences between culture groups was determined by paired Student’s t-tests.

### 2.4 Metagenomic sequencing, assembly, binning and analysis

Metagenomic data for the GT4 samples (four biological replicates) were generated by shotgun sequencing at the Majorbio Bio-Pharm Technology Co. Ltd. Briefly, genomic DNA was randomly broken into fragments at an average size of 400 bp. DNA fragments were end-polished, A–tailed, and ligated with the full-length adapter for paired–end sequencing on Illumina NovaSeq. The paired-end reads were adapter-trimmed, and low-quality reads (length < 50 bp or with a quality value < 20) were removed by fastp (version 0.20.0) (Chen et al., 2018). High-quality reads were subsequently assembled using MEGAHIT (version 1.1.2) (Li et al., 2015). Contigs with a length ≥ 300 bp were retained for downstream analysis. Open reading frames (ORFs) were predicted from the assembled contigs using Prodigal (Hyatt et al., 2010). Predicted ORFs with a length ≥ 100 bp were retrieved and translated into amino acid sequences using Emboss (version 6.6.0) (Rice et al., 2000) and the NCBI translation table.

A non-redundant gene catalog was constructed using CD-HIT version 4.6.1 (Li and Godzik, 2006) with thresholds of 90% sequence identity and 90% coverage. High-quality reads were aligned to the non-redundant gene catalogs with 95% identity using SOAPaligner (Li et al., 2008) to calculate gene abundance. Taxonomic annotation of the non-redundant genes were performed against the NCBI NR database using Diamond version 0.8.35 (Buchfink et al., 2015) with an *e*-value cutoff of 1e^−5^. Functional annotation was predicted against the KEGG database (Kanehisa and Goto, 2000) using Diamond version 0.8.35 with an *e*-value cutoff of 1e^−5^. Gene abundance was normalized using the RPKM (Reads Per Kilobase per Million mapped reads) method.

Metagenome-assembled genomes (MAGs) were constructed using combination of Metabat2 (Kang et al., 2015) (v 2.12.1), MaxBin2 (Wu et al., 2016) (v 2.2.5) and CONCOCT (v 0.5.0) (Alneberg et al., 2014). MAGs with completeness ≥50% and contamination <10% were retained. Taxonomy of the recovered MAGs was determined based on a set of 120 universal single-copy proteins using GTDB-Tk (v 2.3.0) (Parks et al., 2018) against the Genome Taxonomy Database (GTDB).

### 2.5 Quantitative reverse transcription polymerase chain reaction

The expression levels of catechol 1,2-dioxygenase (*catA*) and catechol 2,3-dioxygenase (*C23O*) genes were quantified by reverse transcription quantitative PCR (RT-qPCR). The primer sequences for catA gene were as follows: forward-5′-CCATCTGCATCGGTGA-3′ and reverse-5′-CGTTCGTTSAGCACCCGGTCGTG-3′. For C23O gene, the primers were: forward-5′-GGTCTGATYGAAATGGAYCGCGA-3′ and reverse-5′-CGTTCGTTSAGCACCCGGTCGTG-3′. Detailed protocols, including reaction conditions, and the standard curve method for absolute quantification, are provided in the Supplementary Text S1.

### 2.6 Isolation and characterization of pure bacterial strains

Bacterial strains were isolated from the GT4 enrichment culture. To rejuvenate the enriched consortium while preserving its microbial structure, GT4 was inoculated into MM medium supplemented with 5 g/L crude oil (Charalampous et al., 2021; Gao et al., 2019). Crude oil was added directly without dispersant, and the cultures were incubated at 30 °C with shaking at 150 rpm for 7 days. The bacterial consortia were subjected to serial dilutions (10^−3^ to 10^−5^) in MM medium, and aliquots were spread onto solid MM agar pre-coated with 1 g/L crude oil. The plates were incubated at 30 °C, and colonies with distinct morphologies were selected and subcultured to obtain pure strains. Purified strains were then inoculated into fresh LB medium and cultured at 30 °C with shaking at 150 rpm overnight. Cells were harvested by centrifugation at 8,000 rpm for 5 min and washed twice with sterile saline. The microbial concentrations were adjusted to OD_600_ ~ of 1.0 for subsequent use. The 16S rRNA gene sequences were amplified with universal primers 27F/1492R, and the resulting sequences were analyzed by BLAST against the GenBank database.

To evaluate the crude oil-degrading capacity of enriched consortium and purified strains, cultures (5%, v/v) were inoculated to MM medium supplemented with 5 g/L crude oil and incubated at 30 °C, 150 rpm for 7 days. The uninoculated media served as abiotic controls to account for non-biological loss of hydrocarbons. The residual crude oil was extracted with an equal volume of CH_2_Cl_2_ (dichloromethane), dried in anhydrous sodium sulfate, and concentrated via vacuum rotary evaporation. The concentration of residual crude oil was determined using an infrared oil analyzer, which quantifies hydrocarbons based on the absorption of specific infrared wavelengths by C-H bonds. The components of residual petroleum hydrocarbon after biodegradation were evaluated by gas chromatography (GC) analysis.

### 2.7 Sequence accession numbers

Raw reads of the microbiomes 16S rRNA gene amplicons and the whole-metagenome shotgun sequences of the enrichment consortium have been deposited in the NCBI Sequence Read Archive (SRA) under BioProject accession numbers SRP498344 and SRP498353, respectively.

## 3 Results and discussion

### 3.1 Bacterial community and function prediction of the enrichment cultures

**Figure 1:**
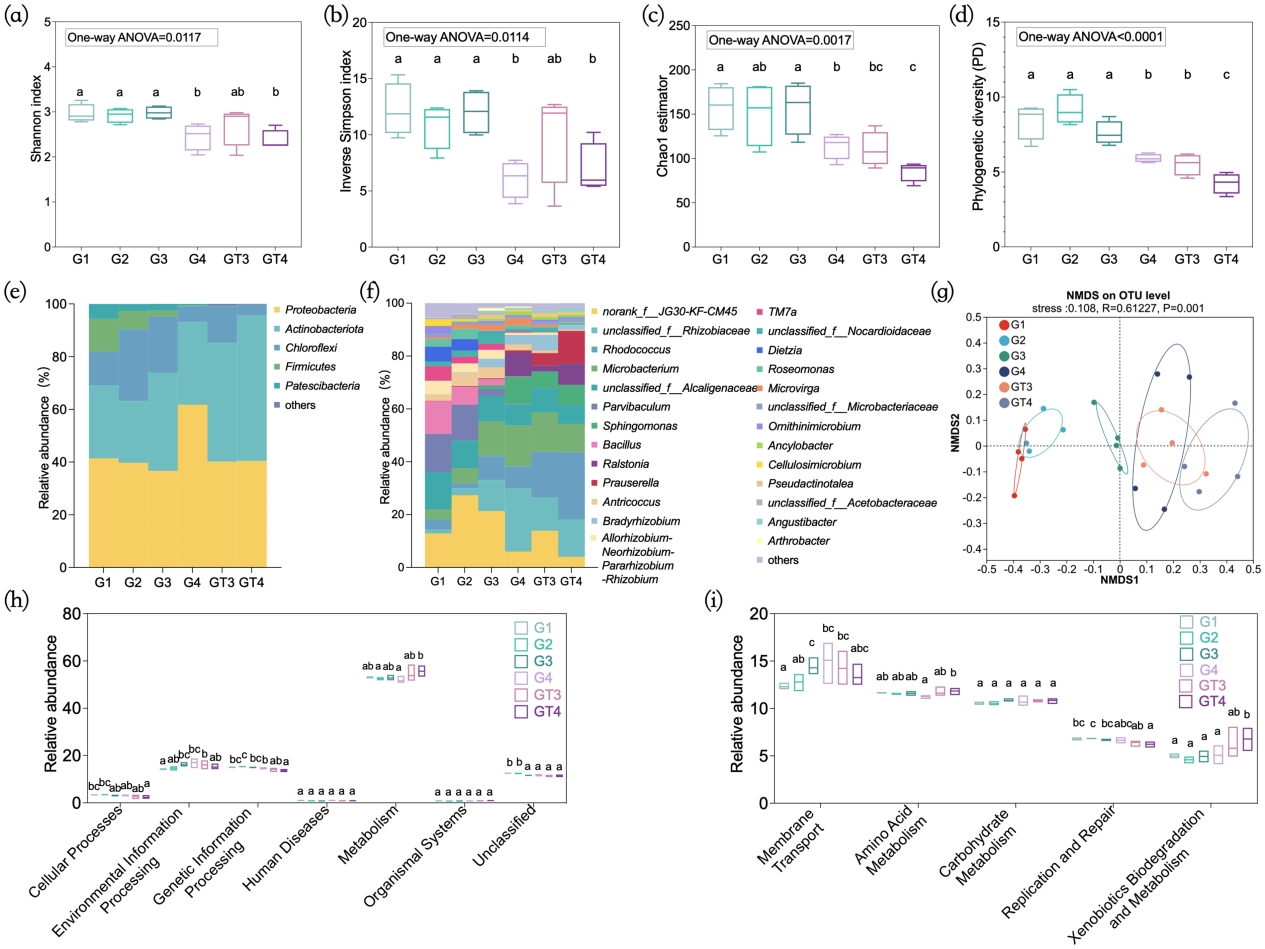
Composition and evolution of the taxonomic and functional profiles of the consortia during the enrichment process. Alpha-diversity indices of Shannon index **(a)**, inverse Simpson index **(b)**, C: Chao1 estimator **(c)**, and phylogenetic diversity **(d)**. Microbial community composition at phylum **(e)** and genus **(f)** level. NMDS analysis of microbial communities on OTU level **(g)**. PICRUSt2 function prediction at KEGG pathway level 1 **(h)** and KEGG pathway level 2 **(i)**. The significance between each group and among the six groups was calculated by one-way ANOVA test, respectively. G1–G4: Four consecutive subcultures with 30-day intervals. GT3–GT4: Prolonged culture samples, obtained by continuous cultivation of G3 and G4 for 90 days.

Shotgun metagenomic sequencing of the four GT4 biological replicates yielded an average of 87.65 million high-quality reads per sample, representing approximately 13.06 Gb of sequence data (Supplementary Table S1). On average, 80,258 contigs per sample were assembled, with a cumulative length of 162.25 Mb. Subsequent gene prediction on the assembled contigs identified a rich genetic reservoir, with an average of 200,535 open reading frames (ORFs) per sample. A total of 1,199,084 sequences were obtained from the 24 enrichment culture samples. After rarefaction to an even depth, each sample contained 25,617 sequences foe downstream analysis. Rarefaction curves indicated sufficient sequencing depth, as all samples reached saturation (Supplementary Figure S2). The alpha-diversity indices for each subculture group were shown in Figure 1. During the serial subculturing phase, the Shannon index, Inverse Simpson index, Chao1 estimator, and Phylogenetic diversity (PD) showed no significant change across the first three transfers (G1-G3). However, all diversity indices declined significantly in G4, which was cultured with a higher crude oil concentration (10 g/L). During prolonged cultivation phase, the Shannon and Inverse Simpson indices of GT3 and GT4 remained relatively stable compared to their, respectively, parent cultures (G3 and G4), whereas the Chao1 and PD values decreased significantly (Figures 1a–d). These results suggest that both increased pollutant concentration and extended incubation time contributed to reduced community complexity. Venn diagram analysis based on OTUs revealed that the unique OTUs decreased substantially throughout the enrichment process, from 10.26% in G1 to zero in GT4 (Supplementary Figure S3).

Crude oil in the culture medium served as the primary selective pressure, allowing only those microorganisms capable of degrading or tolerating the pollutant to persist. To analyze changes in microbial community composition during enrichment, we constructed the total microbial profile at phylum and genus levels for each group. At the phylum level, Proteobacteria, Actinobacteria, and Chloroflexi were dominant, collectively accounting for over 80% of the community throughout the enrichment period. While the relative abundance of *Chloroflexi*, *Firmucutes*, and *Patescibacteria* declined, *Actinobacteria* increased markedly (Figure 1e). At the genus level, bacterial abundances varied considerately across groups (Figure 1f). Several known petroleum hydrocarbon degraders, including *Rhodococcus, Microbacterium, Sphingomonas, Ralstonia, Prauserella,* and *Bradyrhizobium,* were significantly enriched. The relative abundance of *Rhodococcus* and *Microbacterium* increased from 3.75 and 3.85% in G1 to 25.42 and 10.81% in GT4, respectively. Similarly, *Sphingomonas, Ralstonia* and *Prauserella,* which initially represented less than 0.2% of the community in G1, increased to 7.68, 7.99, and 12.42% in GT4, respectively. In contrast to enrichment results from the Yanchang oilfield in northern Shaanxi, China (Yu et al., 2022), hydrocarbon-degrading genera such as *Bacillus*, *Parvibaculum,* and *Dietzia,* were depleted in this study.

Differences in bacterial community composition across different groups were illustrated by NMDS analysis based on the OTU distribution (stress = 0.108, *R* = 0.610). The results revealed a gradual shift in community structure throughout the enrichment process (Figure 1g). The microbial community in G3 was distinct from the initial community in G1, and G4 was further separated from G3, indicating that sequential enrichment with increasing oil concentration significantly altered community structure (Táncsics et al., 2023; Yu et al., 2022). Furthermore, the prolonged cultures (GT3 and GT4) were separated from their parent cultures (G3 and G4), respectively, indicating that extended cultivation time also shaped community composition (Ma et al., 2020). Phylogenetic diversity of degrading genera showed no significant differences among the groups (One-way ANOVA, *p* = 0.1689) (Supplementary Figure S4). These results indicated that the enrichment strategy served as a filter that selected and maintained a phylogenetically coherent core of degraders.

The functional potential of bacterial communities was predicted using PICRUSt2 based on KEGG pathway annotations. As shown in Figure 1h, the majority of predicted genes were associated with metabolism (53.4%), environmental information processing (15.5%), and genetic information processing (14.6%). The relative abundances of genes in metabolism and genetic information processing categories showed no significant change from G1 to G4. Similarly, no significant differences were observed for these three metabolic categories between GT3 and G3 during prolonged cultivation. However, in GT4 compared to G4, metabolism functions increased significantly, genetic information processing decreased, and environmental information processing remained unchanged.

Further analysis at KEGG pathway level 2 revealed that the dominant pathways included membrane transport (13.6%), amino acid metabolism (11.6%), carbohydrate metabolism (10.7%), replication and repair (6.6%), and xenobiotics biodegradation and metabolism (5.4%) (Figure 1i). The relative abundances of these pathways remained relatively stable during the serial subculturing phase (G1-G4) and showed no significant differences between GT3 and G3. Notably, in GT4 compared to G4, amino acid metabolism and xenobiotics biodegradation and metabolism pathways were significantly enhanced. It is worth noting that the PICRUSt2 predictions for GT4 toward “xenobiotics biodegradation and metabolism” function showed a strong and statistically significant correlation (Spearman’s *r* = 0.939, *p* < 0.0001) with the shotgun metagenomic data (Supplementary Figure S5). This strong concordance indicated that the PICRUSt2 predictions reliably captured the genuine functional potential of the microbial communities, particularly for the core metabolic pathways under investigation.

These results suggested that increasing selective pressure combined with long-term acclimation was key to driving the structural simplification and functional enhancement. This finding is consistent with the role of selective pressure in enriching functional microorganisms (Marietou et al., 2018; Vigneron et al., 2021). While a reduction in community diversity is often considered a risk to stability, in GT4, the relative abundance of core genera (such as *Microbacterium*) and metabolic potential significantly increased. This suggested that in engineering microbial consortia, efforts should focus on constructing a streamlined core consortium composed of highly efficient and complementary strains, which is crucial for improving its predictability and performance during in-situ remediation (Spini et al., 2018).

### 3.2 Functional genes and microorganisms in consortia GT4

**Figure 2:**
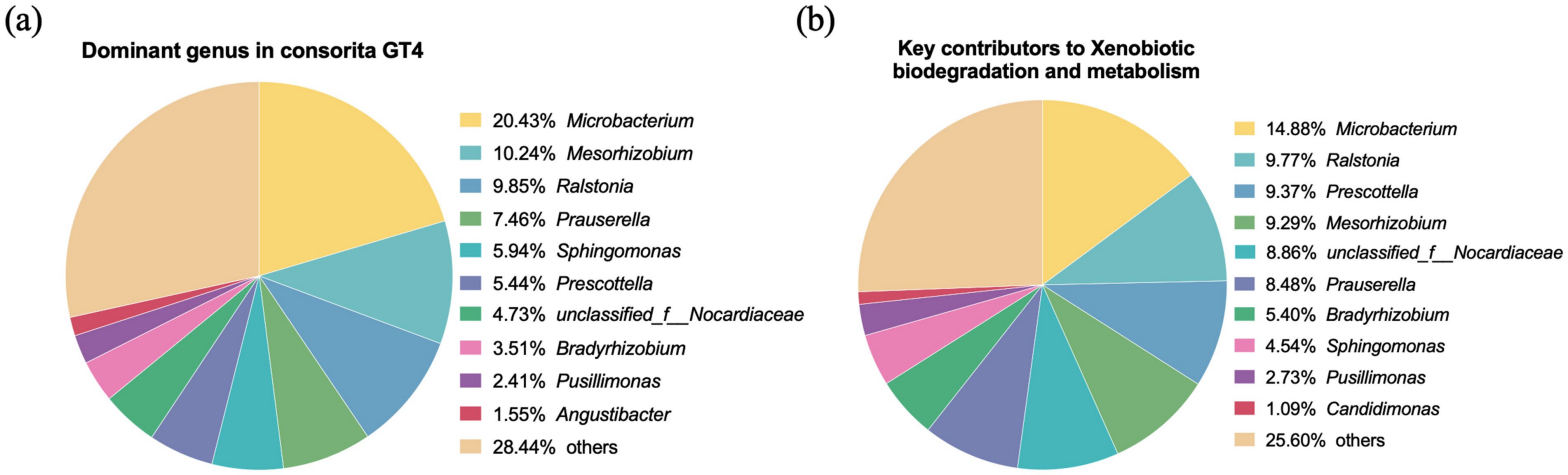
Metagenomic analysis revealed the core contributors related to hydrocarbon degradation of consortia GT4. Pie chart showing the relative abundance of the dominant bacterial genera (≥1%) in the GT4 consortia based on metagenomic analysis **(a)**. The key genera (≥1%) annotated under the “xenobiotic biodegradation and metabolism” pathway (KEGG level 2) based on metagenomic sequencing **(b)**. The “others” category aggregates all genera with a relative abundance below 1%. Specific percentages for each genus are annotated on the charts.

Given that GT4 exhibited high degradation potential with a simplified structure, metagenomic analysis was applied to identify the functional genes and metabolic pathways for hydrocarbon degradation and to investigate the intricate microbial interaction network within this consortia. As presented in Figure 2a, genera with a relative abundance above 1% included *Microbacterium, Mesorhizobium, Ralstonia, Prauserella, Sphingomonas, Prescottella,* unclassified*_Nocardiaceae, Bradyrhizobium, Pusillimonas,* and *Angustibacter*. To identify the core taxa contributing to hydrocarbon degradation, functional contribution analysis was conducted at KEGG pathway level 3. The “xenobiotic biodegradation and metabolism” pathway was among the most abundant functions, with major contributions from *Microbacterium, Ralstonia, Prescottella, Mesorhizobium,* unclassified*_Nocardiaceae, Prauserella, Bradyrhizobium, Sphingomonas,* and *Pusillimonas* (Figure 2b). These key functional contributors corresponded exactly to the dominant genera in GT4, confirming that the top ten dominant genera constitute the core crude oil-degrading taxa. These results indicated that the enrichment strategy effectively selected for microorganisms with key functional roles in hydrocarbon degradation.

Under aerobic conditions, monooxygenases (including hydroxylases) and dioxygenases are the key enzymes responsible for the initial oxidation of hydrocarbons (Dallinger et al., 2016; Li et al., 2020; Phale et al., 2019; Rojo, 2009). In the GT4 consortia, a total of 345 coding DNA sequences (CDSs) were annotated as oxygenases involved in the initial degradation of alkanes and aromatic compounds (Supplementary Table S2). These CDSs were assigned to 86 genera, among which 7 genera contained more than 10 CDSs: *Agromyces* (12 CDSs), *Arthrobacter* (10 CDSs), *Microbacterium* (105 CDSs), *Microvirga* (11 CDSs), *Nocardia* (13 CDSs), *Prauserella* (16 CDSs), and *Ralstonia* (11 CDSs) (Supplementary Figure S6). Specifically, 79 CDSs were involved in alkane degradation, corresponding to 15 alkane 1-monooxygenase (alkM) and 64 long-chain alkane monooxygenase (ladA) genes. Fifty-eight CDSs were identified that initiate degradation of benzene, toluene, chlorobenzene, naphthalene, and phenol. These comprised 1 CDS for the benzene/toluene/chlorobenzene dioxygenase ferredoxin component (todB), 3 CDSs for naphthalene 1,2-dioxygenase component (nahAa and nahAb), 54 CDSs for phenol 2-monooxygenase (E1.14.13.7). Furthermore, 208 CDSs were detected for degrading the central intermediates of aromatic hydrocarbons. These included benzoate/toluate 1,2-dioxygenase (14 CDSs), catechol 1,2-dioxygenase (13 CDSs), catechol 2,3-dioxygenase (92 CDSs), protocatechuate 3,4-dioxygenase (41 CDSs), protocatechuate 4,5-dioxygenase (6 CDSs), homogentisate 1,2-dioxygenase (22 CDSs), phthalate 4,5-dioxygenase (14 CDSs), and p-cumate 2,3-dioxygenase (6 CDSs).

Quantitative reverse transcription PCR (RT-qPCR) was conducted to access the expression levels of the key genes. The RT-qPCR result showed the copy numbers of 114 ± 16 and 3,264 ± 523 copies/ng DNA for catechol 1,2-dioxygenase and catechol 2,3-dioxygenase, respectively, with correspondent Ct value of 31 and 25 (Supplementary Table S3). This result was highly consistent with the metagenomic data, which showed a substantial greater number of CDSs for catechol 2,3-dioxygenase (92 CDSs) than for catechol 1,2-dioxygenase (13 CDSs). This result confirmed that oil metabolism proceeded via the proposed genes, thereby validating the aromatic compound degradation capacity of the GT4 consortia.

**Figure 3:**
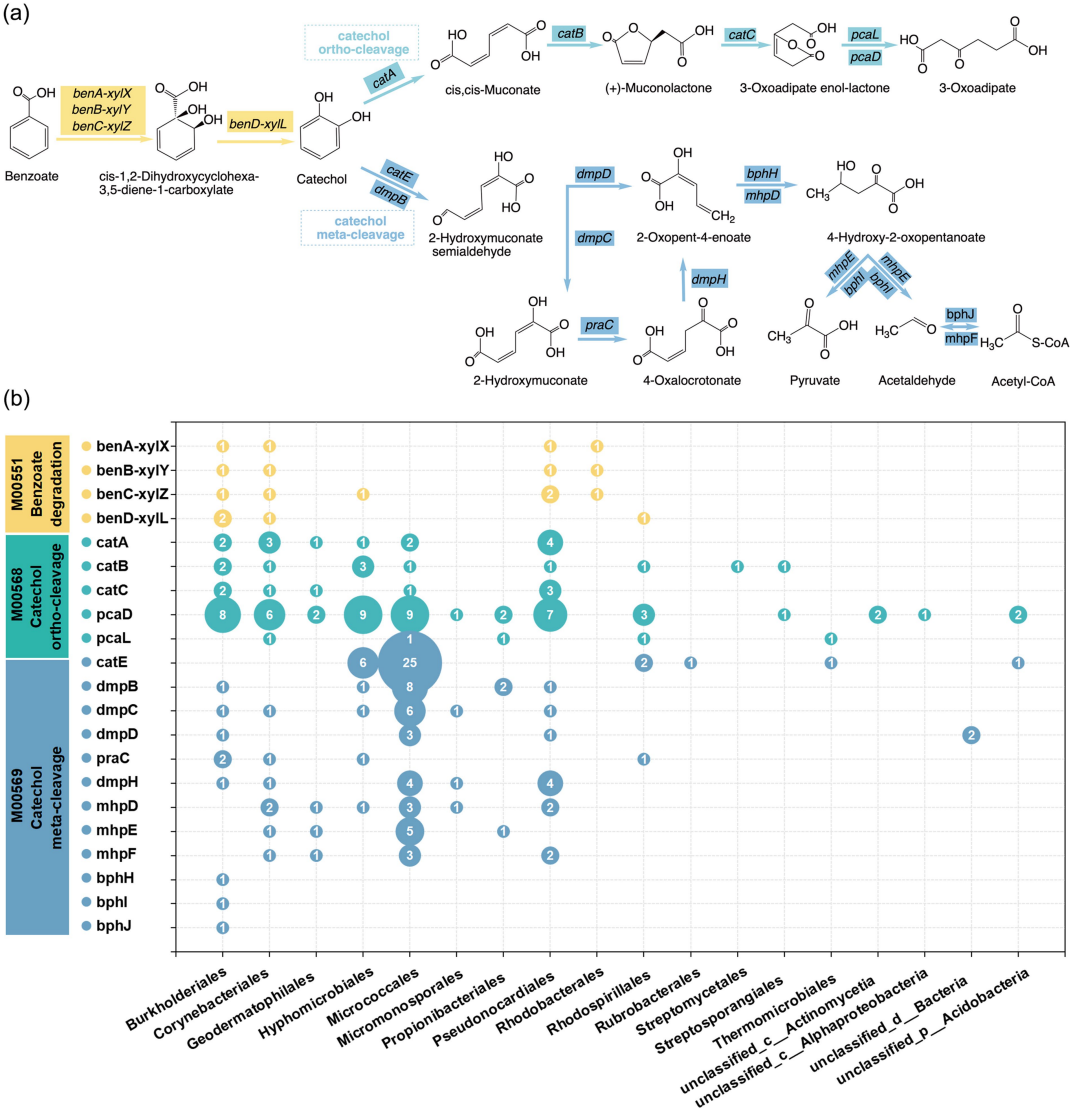
Metagenomic analysis revealed the vital contributors related to the benzoate and catechol degradation pathways. The transformation of benzoate to catechol and the ortho-cleavage and meta-cleavage pathways of catechol **(a)**. Number of identified CDSs related to the degradation pathways of benzoate and catechol **(b)**.

Benzoate and catechol are central intermediates in aromatic hydrocarbon degradation (Eze et al., 2021; Wu et al., 2023). The GT4 metagenome contained a full suite of CDSs predicted to support complete degradation of both compounds (Figure 3a). A total of 219 CDSs were involved in the benzoate and catechol degradation. These CDSs were assigned to 72 genera across 18 orders. Twelve genera contained more than five CDSs, including the *Microbacterium*, *Prauserella*, *Ralstonia*, *Prescottella*, *Ornithinimicrobium*, *Agromyces*, *unclassified_o__Burkholderiales*, *Roseomonas*, *Nocardioides*, *Nocardia*, *Ancylobacter,* and *Bradyrhizobium* (Supplementary Figure S7). In the benzoate degradation pathway, the benzoate/toluate 1,2-dioxygenase (benA-xylX, benB-xylY, and benC-xylZ) and dihydroxycyclohexadiene carboxylate dehydrogenase (benD-xylL) convert benzoate into catechol and methylcatechol (Parales et al., 2008). In GT4, 18 CDSs involved in benzoate degradation were assigned to 6 taxonomic orders, with *Burkholderiales* and *Corynebacteriales* harboring all four key genes (Figure 3b). Catechol degradation proceeds through two major routes: the ortho-cleavage and meta-cleavage pathways (Fuchs et al., 2011; Habe and Omori, 2003; Nešvera et al., 2015; Parales et al., 2008). In GT4, 90 CDSs affiliated to 15 orders participated in the catechol ortho-cleavage pathway. Among them, *Pseudonocardiales* contributed the most (15 CDSs), followed by *Burkholderiales* and *Micrococcales* (14 CDSs each) (Figure 3b). Genes involved in the catechol meta-cleavage pathway were *catE*, *todF*, *dmpBCDH*, *mhpDEF*, *bphHIJ*, and *praC*, which accounted for 111 CDSs across 13 orders in GT4. Micrococcales stood out with 57 CDSs, of which 33 encoded dioxygenase *catE* and *dmpB* (Figure 3b).

MAG analysis was performed to link the functional potential with specific bacterial lineages at a higher resolution. GT4 consortia generated 44 non-redundant, medium- or high-quality MAGs. The completeness and contamination statistics for all 44 MAGs were provided in Supplementary Table S4. The MAGs were classified into 25 distinct genera. Notably, the genus *Microbacterium* was overwhelmingly dominant, represented by 15 MAGs (34.1% of the total). This genomic evidence solidifies *Microbacterium* as a keystone genus within the GT4 consortia. Key oxygenase genes and their affiliated species were analyzed in these MAGs (Supplementary Figure S8). These MAGs encoded a complete repertoire of genes involved in the degradation pathways of benzoate, catechol and phenol. Especially, we found *Prauserella muralis* (MAG31) possessed the entire gene complement for catechol degradation. These results provided a mechanistic, genome-resolved explanation for the strong functional signal demonstrated by metagenomic analyses.

Metagenomic analysis not only confirmed the core status of known degraders but, more importantly, systematically revealed the complete aromatic hydrocarbon degradation pathways they carry (e.g., benzoate, catechol pathways). Unlike the consortium enriched by Yu et al. from Xinjiang oil field (dominated by *Dietzia*) (Yu et al., 2022), GT4 consortia were dominated by *Microbacterium*. This compositional difference can likely be attributed to the distinct selective conditions employed. The discovery of a complete catechol degradation pathway in the *Prauserella muralis* (MAG31) genome suggested a key role in aromatic hydrocarbon degradation. Despite the absence of direct experimental evidence for *P. muralis*, the confirmed hydrocarbon-degrading phenotypes of its relatives, *P. soli* (Almutairi, 2015) and *P. oleivorans* (Dastgheib et al., 2017), provided robust phylogenetic support for this inferred function. This pattern implied that hydrocarbon degradation might be a conserved trait in the genus, highlighting how metagenomics can uncover the ecological roles of specific taxa and identify novel degraders like *P. muralis*.

### 3.3 Key biogeochemical cycles in consortia GT4

**Figure 4:**
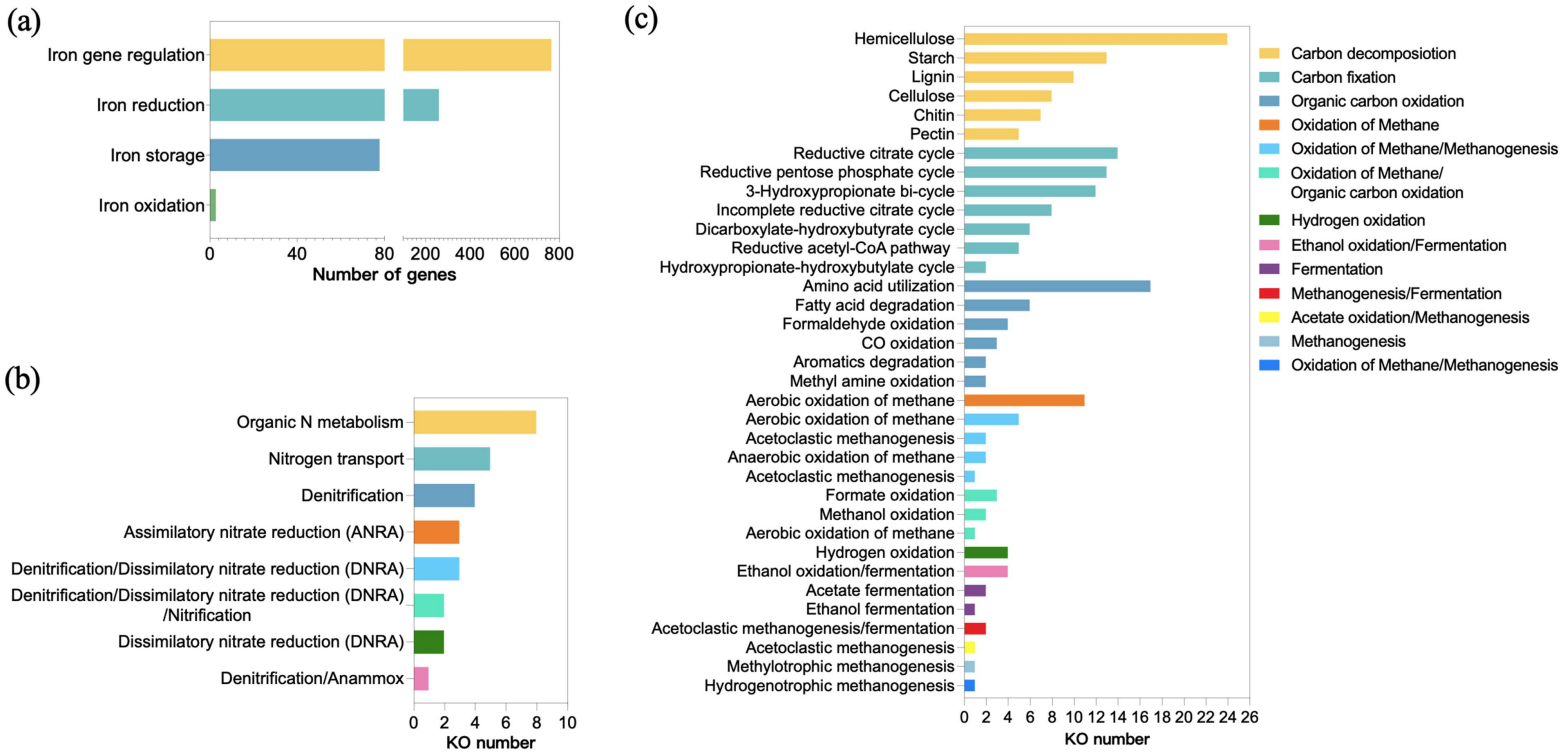
Analysis of key biogeochemical cycles in consortia GT4. Abundance of genes involved in iron metabolism **(a)**. Discontinuous *x*-axis was used with segments spanning 0–100 and 200–800. The KEGG ortholog (KO) numbers for nitrogen metabolism **(b)** and carbon metabolism **(c)**.

Iron plays an important role in hydrocarbon degradation. The majority of the redox enzymes involved in hydrocarbon biodegradation contain iron (Laczi et al., 2015). The keystone alkane degradation enzyme AlkB relies on Fe(II) at its active site to activate molecular oxygen (Guo et al., 2023; Tsai et al., 2017), enabling terminal hydroxylation of alkanes. Iron (III) serves as an electron acceptor, facilitating the degradation of organic matter. The metabolic potential for anaerobic hydrocarbon degradation coupled to Fe(III) reduction has also been demonstrated (Di et al., 2025; Ning et al., 2018). Thus, Fe metabolic analysis was conducted to study the iron metabolism within GT4 community. The results revealed that the detected iron metabolism genes were primarily involved in iron gene regulation (69.7%), iron reduction (23.9%), iron storage (5.6%), and iron oxidation (0.8%) (Figure 4a). This indicated the microbial community possessed a sophisticated iron homeostasis regulatory capacity, which may be essential for both aerobic and anaerobic enzymatic transformations of hydrocarbons.

The key pathways for nitrogen and carbon metabolisms were also analyzed. Crude oil is carbon-rich but nitrogen-deficient, making nitrogen availability a critical factor limiting microbial growth and hydrocarbon biodegradation rates (Kappell et al., 2014). The GT4 community exhibited enriched pathways for organic nitrogen metabolism and transport (Figure 4b). This capability likely provided a significant competitive advantage by enabling the synthesis of nitrogen-rich enzymes and biomass required for proliferation on oily substrates (Sarkar et al., 2016; Smith et al., 2013). The diverse central carbon metabolism (Figure 4c), encompassing the carbon decomposition, carbon fixation and organic carbon oxidation pathways, indicated a highly integrated and efficient carbon-processing system (Vigneron et al., 2021). This diversity might allow the community to mineralize a broad spectrum of carbon sources. Specifically, the presence of carbon fixation pathways (e.g., the reductive acetyl-CoA pathway), coupled with hydrogen oxidation pathway, suggested the potential for autotrophic CO_2_ assimilation (Figure 4c) (Hattori, 2008). These results indicated both aerobic and anaerobic metabolisms in GT4 consortia, showing its adaptation to thrive in long-term, oil-rich environments and provided a genomic basis for its degradation ability.

### 3.4 Correlation between bacterial phylogeny and function

**Figure 5:**
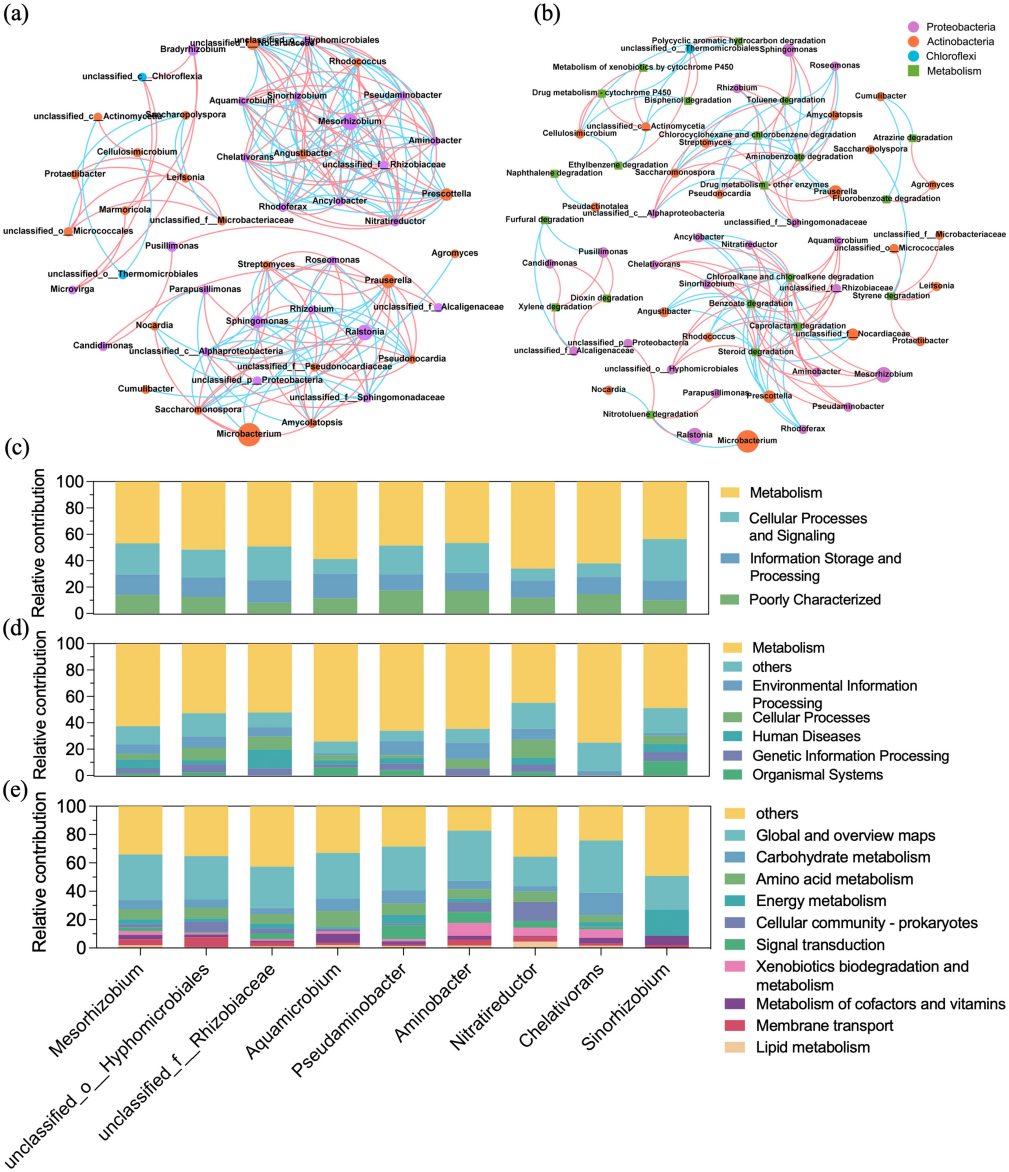
Co-occurrence analysis of GT4 and the function contribution of the positive correlated nine diazotrophic genera. Co-occurrence network between microbial taxa **(a)** and between function and microbial taxa **(b)**. The circle nodes represent the microbial taxa (labeled at genus level, colored at phylum level), and squares represent function (xenobiotic biodegradation and metabolism). The edges represent a significantly strong correlation (Spearman’s rank correlation: |*r*| > 0.85, *p* < 0.01), and pink and blue edges denote positive and negative correlation, respectively. Species and functional contribution analysis of the nine diazotrophic genera at COG category **(c)**, KEGG level 1 **(d)**, and KEGG level 2 **(e)**.

To uncover co-occurrence patterns among genera in GT4, the 50 most abundant genera were selected for correlation network analysis. The resulting network comprised 48 nodes and 193 edges and naturally clustered into eight distinct modules. The two largest modules collectively contained 26 nodes. Each module showed dense intra-module connections, suggesting potential functional or ecological interactions among the constituent taxa. As depicted in Figure 5a, the genus *Mesorhizobium* possessed the highest centrality score (0.298) and the highest number of positive correlations, indicating its potential keystone role in the microbial community. Genera positively associated with *Mesorhizobium* included unclassified *Hyphomicrobiales,* unclassified *Rhizobiaceae, Aquamicrobium, Pseudaminobacter, Aminobacter, Nitratireductor, Chelativorans,* and *Sinorhizobium*. Interestingly, the majority of these associated taxa were known diazotrophic bacteria. The synergistic partnership of these diazotrophic bacteria might alleviate nutritional restriction and maintain the stability of the microbial community (Chaudhary et al., 2019). Species and functional contribution analysis confirmed that these nine diazotrophic genera contributed mostly to the core metabolic pathways (Figures 5c,d), especially carbohydrate metabolism, amino acid metabolism, energy metabolism, and xenobiotics biodegradation and metabolism (Figure 5e). Their roles in xenobiotics degradation was consistent with previous studies characterizing these genera as known hydrocarbon degraders (Ellegaard-Jensen et al., 2017; Keum et al., 2006; Ma et al., 2021; Topp et al., 2000; Xu et al., 2017; Yu et al., 2023). This function overlap underpinned their potential for synergistic biodegradation in the consortia. Collectively, these findings suggested strong synergistic interactions among community members and highlighted the pivotal role of diazotrophic bacteria in crude oil biodegradation.

To investigate correlation between microbial taxa and hydrocarbon degradation functions, a co-occurrence network was established based on the top 50 genera and level 2 KEGG pathways within “xenobiotic biodegradation and metabolism,” with a strong correlation of Spearman’s *r* > 0.85, *p* < 0.01. As exhibited in Figure 5b, four degradation functions, benzoate degradation, caprolactam degradation, chloroalkane and chloroalkene degradation, and steroid degradation were centrally located within module I, with a centrality score of 0.234. Genera positively correlated with these functions included *Prescottella,* unclassified_*Nocardiaceae, Angustibacter, Ancylobacter, Rhodococcus*, and *Rhodoferax*. Three genera played the pivotal roles in module II: *Cellulosimicrobium,* unclassified*_Thermomicrobiales* and unclassified*_Actinomycetia.* Each exhibited a centrality score of 0.078. These genera demonstrated positive correlations with eight degradation functions: polycyclic aromatic hydrocarbon degradation, ethylbenzene degradation, bisphenol degradation, drug metabolism-cytochrome P450, metabolism of xenobiotics by cytochrome P450, polycyclic aromatic hydrocarbon degradation, ethylbenzene degradation, and bisphenol degradation. These results revealed that the certain genera positively correlated with hydrocarbon degradation functions, suggesting their biodegradation potential and informing the rational design of synthetic microbial consortia.

### 3.5 Biodegradation of crude oil by the consortium and isolated strains

**Figure 6:**
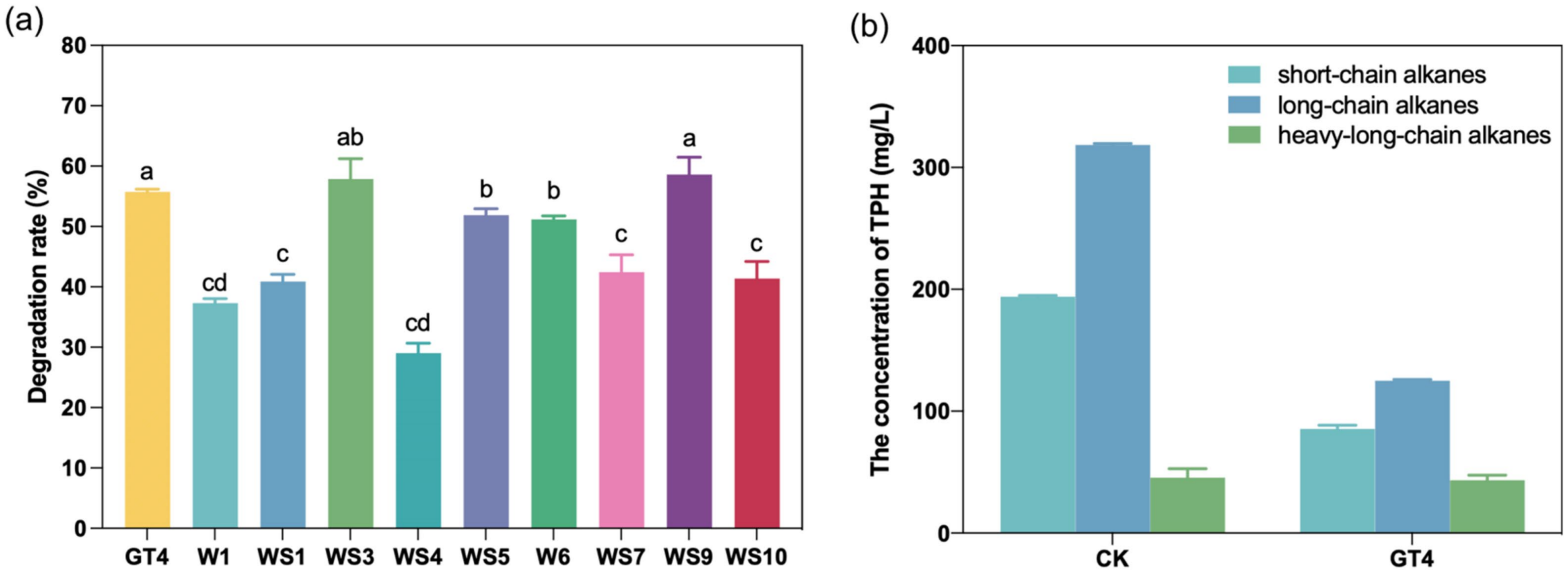
Crude oil degradation abilities of nine purified strains compared with consortia GT4. The crude oil-degrading ability of GT4 and nine purified strains **(a)**, and degradation of petroleum hydrocarbon with different chain length in crude oil by GT4 **(b)**.

A total of nine strains were isolated from the GT4 enrichment culture. The strains were identified as *Bacillus velezensis* W1, *Caulobacter* sp. WS1, *Microbacterium algeriense* WS3, *Peribacillus frigoritolerans* WS4, *Cellulosimicrobium aquatile* WS5, *Paracandidimonas caeni* W6, *Cellulosimicrobium* sp. WS7, *Cellulosimicrobium cellulans* WS9, and *Prescottella equi* WS10 (Supplementary Figures S9a–i). Among these, strains WS3 and WS9 showed the strongest capacity to degrade crude oil at the initial concentration of 5 g/L, with degradation rate of 57.85 and 58.60%, respectively. These rates were not significantly different from that of GT4 consortia (Figure 6a). The biodegradation ability to n-alkanes (C_10_–C_40_) was evaluated by semi-quantitative analysis of GC data for residual versus abiotic controls. According to previous studies (Li et al., 2013), n-alkanes were categorized into short-chain (C10-C17), long-chain (C18-C30), and heavy long-chain (C31-C38) fractions. As shown in Figure 6b, the GT4 consortia exhibited strong degradation of both short-chain and long-chain alkanes, with degradation ratios of 55.98 and 60.70%, respectively. The correspondent GC chromatograms were displayed in Supplementary Figure S10.

Top-down enrichment, guided by evolutionary principles, improves the trait of interest and generates high-performance community, from which potent strains with extraordinary degradability are explored (Chen et al., 2023; Ma et al., 2020; Stari et al., 2023; Yu et al., 2022). In this study, nine strains affiliated with seven genera were isolated and characterized, including the widely recognized hydrocarbon-degrading genera *Microbacterium* and *Bacillus* (Kim et al., 2018). Notably, five of the nine strains belonged to low-abundance genera (relative abundance < 1%) in the GT4 consortia, such as *Cellulosimicrobium, Paracandidimonas,* and *Caulobacter*. Despite their low abundance in the consortia, two newly isolated *Cellulosimicrobium* strains (WS5 and WS9) degraded more than 50% of the crude oil (Figure 6a). Although isolates from low-abundance taxa demonstrated high degradation potential in pure culture, their actual *in situ* contribution within the consortium may differ due to community interactions and metabolic division of labor. Further validation (e.g., through co-culture assays or metatranscriptomic analysis) would be needed to determine their ecological role within GT4.

The genus *Cellulosimicrobium,* belonging to the *Actinomycetes*, is known for its tremendous biodegradation capacity for various xenobiotic pollutants in soil and aquatic environments (Srivastava et al., 2022). Members of *Cellulosimicrobium* have been isolated and characterized as hydrocarbon degraders. Shaieb et al. showed the *C. cellulans* could decrease 18.9% of crude oil concentration in 15 days in batch culture experiments (Shaieb et al., 2015). Nkem et al. demonstrated the 2% (w/v) diesel oil degradability of *C. cellulans* was 64.4% within 10 days in a seawater medium (Nkem et al., 2016). Qin et al. Reported that *C. cellulans* CWS2 was able to degrade benzo(a)pyrene under anaerobic condition (Qin et al., 2018).

It should be noted that the crude oil-degrading capacity of the other two minor genera (*Paracandidimonas and Caulobacter*) had not been experimentally validated prior to this study. *Paracandidimonas* was first isolated from soil in Alabama, United States in 2017 (Kämpfer et al., 2017). Few *Paracandidimonas* species have been isolated and characterized, and their specific ecological functions remained poorly defined. *Paracandidimonas caeni* 24, a type strain isolated from activated sludge, was reported to degrade herbicide metoprolol (Yao et al., 2019). Members of the genus *Caulobacter* have been isolated from both aquatic and terrestrial ecosystems. However, most efforts have focused on their adaptation to aquatic, oligotrophic environments, while the ecology and function of *Caulobacter* in non-aquatic or nutrient-rich environments have received little attention (Wilhelm, 2018). However, recent studies have begun to uncover their presence and ecological roles in terrestrial environments, including the degradation of cellulose (Verastegui et al., 2014; Wang et al., 2007; Wilhelm et al., 2017), lignin (DeAngelis et al., 2011), and petroleum hydrocarbons (Corgié et al., 2004; Li Y. J. et al., 2022; Yergeau et al., 2012). In this study, *Paracandidimonas caeni* W6 and *Caulobacter.* sp. WS1 demonstrated crude oil degradation efficiencies of 51.19 and 40.90%, respective (Figure 6a). To the best of our knowledge, this study provides the first experimental evidence supporting the crude oil-degrading capacities of the genera *Paracandidimonas* and *Caulobacter.* This finding expanded the known diversity of hydrocarbon-degrading bacteria.

## 4 Conclusion

This study demonstrated how selective pressures shaped highly efficient crude oil-degrading consortia through a top-down enrichment strategy. The combination of elevated substrate concentration and prolonged cultivation was pivotal in driving the microbial community toward structure simplification and functional enrichment. This process significantly reduced phylogenetic diversity while concurrently enriching key degraders and enhancing the predicted potential for xenobiotic metabolism, ultimately yielding the simplified yet high-performance GT4 consortia. Metagenomic and MAGs analyses not only confirmed the central role of genera like *Microbacterium* in desired degradation pathways but also, via co-occurrence network analysis, revealed extensive potential syntrophic interactions between diazotrophic bacteria and hydrocarbon degraders. The successful isolation of novel degraders, including *Paracandidimonas* and *Caulobacter*, underscored the effectiveness of the top-down enrichment approach in accessing underexplored microbial resources. The GT4 consortium itself and the isolated high-efficiency strains represented promising inoculants for bioremediation in crude oil-contaminated environments. The insights into the synergistic interactions within the consortia provide a rational basis for designing synthetic microbial communities with enhanced robustness, thereby improving the predictability and efficiency of field-scale bioremediation.

## Data Availability

The data presented in this study are publicly available. The data can be found here: https://www.ncbi.nlm.nih.gov/sra, accession SRP498344 and SRP498353.
