# Supplementary material for: Top-down enrichment of oil-degrading microbial consortia reveals functional streamlining and novel degraders
Source: Front Microbiol. 2025 Dec 3;16:1656448. doi: 10.3389/fmicb.2025.1656448 (PMC12711144; doi:10.3389/fmicb.2025.1656448)
Supplement: Supplementary file 1 [file Data_Sheet_1.docx]

**Supporting Information**

**Top-Down Enrichment of Oil-Degrading Microbial Consortia Reveals Functional Streamlining and Novel Degraders**

*Huijun Wu^1^, Xianyuan Du^1^, Jin Zheng^1^, Xingchun Li^1^, Quanwei Song^1^, Yuhao Yan^1, 2^, Anzhou Ma^3^, Anming Xu^4^, Jufeng Li^1*^*

*^1^State key laboratory of petroleum pollution control, China National Petroleum Corporation research institute of safety and environmental technology, Beijing 102206, P. R. China*

*^2^College of Environmental Science and Engineering, Shaanxi University of Science & Technology, Xi’an, 710021, P. R. China*

^3^*University of the Chinese Academy of Sciences, Beijing 100049, China*

^4^*College of Biotechnology and Pharmaceutical Engineering, Nanjing Tech University, Nanjing, 211816, China*

*Corresponding authors.

Jufeng Li: [lijufeng69@163.com](mailto:ljf69@petrochina.com.cn;)

State Key Laboratory of Petroleum Pollution Control, China National Petroleum Corporation Research Institute of Safety & Environment Technology, Beijing 102206, P. R. China.

**Contents: 1 text, 10 figures and 4 tables.**

**Text S1.** The expression levels of catechol 1,2-dioxygenase (*catA*) and catechol 2,3-dioxygenase (*C23O*) genes were quantified by reverse transcription quantitative PCR (RT-qPCR). Total RNA of GT4 samples were extracted using Quick-RNA Fecal/Soil Microbe Microprep Kit (Zymo Research, USA). Reverse transcription of the total RNA was performed with HiScript 1st Strand cDNA Synthesis Kit (Vazyme, China) to obtain cDNA with the following thermocycling conditions: 5 min at 25 ℃ followed by 15 min at 50 ℃ and then 5 min at 85 ℃. The qPCR reactions were performed on a CFX96 Touch Real-Time PCR Detection System (Bio-Rad, USA). AceQ qPCR SYBR Green Master Mix (Vazyme, China) was used. Each reaction was carried out in a total volume of 15 μL, containing 7.5 μL of 2×SYBR Green Mix, 0.7 μL of each forward and reverse primer (10 μM), 1 μL of diluted cDNA template, and 5.8 μL of nuclease-free water. The primer sequences for catA gene were as follows: forward-5’- CCATCTGCATCGGTGA-3’ and reverse-5’- CGTTCGTTSAGCACCCGGTCGTG-3’. For C23O gene, the primers were: forward-5’- GGTCTGATYGAAATGGAYCGCGA-3’ and reverse-5’-CGTTCGTTSAGCACCCGGTCGTG-3’. The qPCR program was as follows: denaturation at 95 ℃ for 15 s, followed by 45 cycles of 15 s at 95 ℃, 15s at 55 ℃, and 35 s at 72 ℃. A melt curve analysis was conducted at the end of each run to confirm the specificity of the amplification, by heating 15 s at 95 °C, 60 s at 65 °C, 30 s at 95 °C, and 15 s at 95 °C. Three biological replicates and three technical replicates were performed. For absolute quantification, the Ct value was substituted into the standard curve equation to obtain the logarithm of the copy number, which was then converted to the absolute copy number by raising 10 to the power of the resulting value.

**Figures**

**Fig. S1.** Schematic of enrichment strategy.

**Fig. S2.** The rarefaction curves of each consortium sample.

**Fig. S3.** The Venn plot showing the shared and unique OTUs between G1–GT4.

**Fig. S4.** Phylogenetic diversity of degrading microbes of the enrichment consortia.

**Fig. S5.** Concordance between PICRUSt2 prediction and shotgun metagenomics for xenobiotics biodegradation and metabolism pathway Each point represents a degradation pathway. The blue line shows linear regression with the 95% confidence interval. Statistical significance was assessed by Spearman correlation.

**Fig. S6.** Metagenomic analysis revealed key oxygenases and affiliated genera involved in alkane and aromatic compounds degradation. Only genera harboring more than five CDSs are displayed.

**Fig. S7.** Metagenomic analysis revealed the genera contained over five CDSs of benzoate and catechol degradation pathways.

**Fig. S8.** Genomic potential for hydrocarbon degradation reconstructed from MAGs. Key oxygenases and the species involved in alkane and aromatic compound degradation are displayed.

**Fig. S9.** Phylogenetic tree of nine purified strains: W1 (a), WS1 (b), WS3 (c), WS4 (d), WS5 (e), W6 (f), WS7 (g), WS9 (h), WS10 (i).

**Fig. S10.** Gas chromatogram (GC) of biodegradation efficiency of saturated hydrocarbons in crude oil by consortium treatment in 7 days. The sterial abiotic control (a) and GT4 consortium treatment (b).

**Tables**

**Table S1.** Metagenomic assembly statistics for the four GT4 biological replicates.

**Table S2.** Key oxygenase genes and affiliated genera involved in the degradation of alkanes and aromatic compounds.

**Table S3.** Quantitative RT-PCR results for key aromatic degradation genes in the GT4 consortia.

**Table S4.** The completeness and contamination statistics for all MAGs in GT4.
