# Supplementary figures and images for: Top-down enrichment of oil-degrading microbial consortia reveals functional streamlining and novel degraders
Source: Front Microbiol. 2025 Dec 3;16:1656448. doi: 10.3389/fmicb.2025.1656448 (PMC12711144; doi:10.3389/fmicb.2025.1656448)

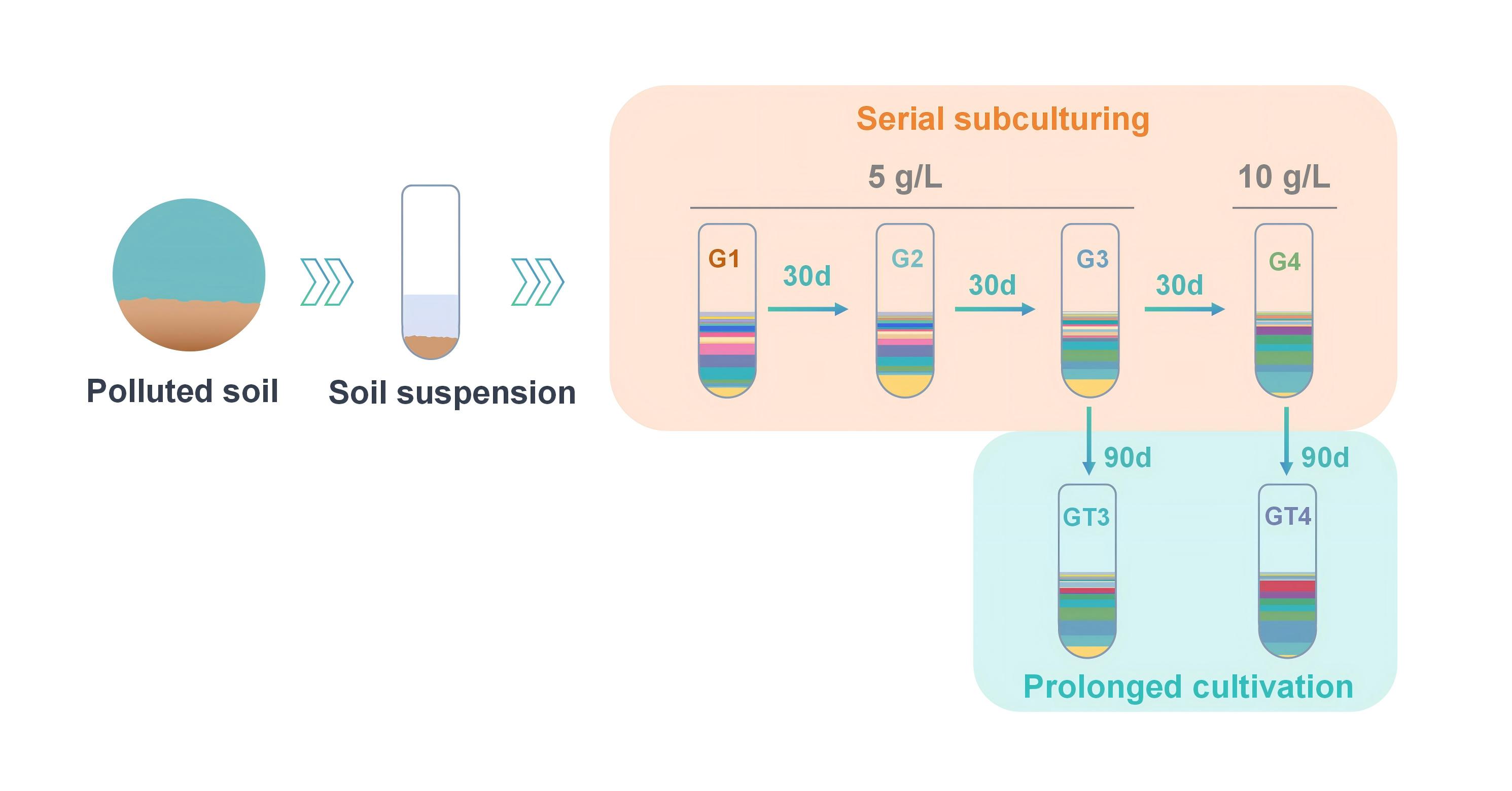

Supplement: Supplementary file 2 [file Image_1.jpeg]

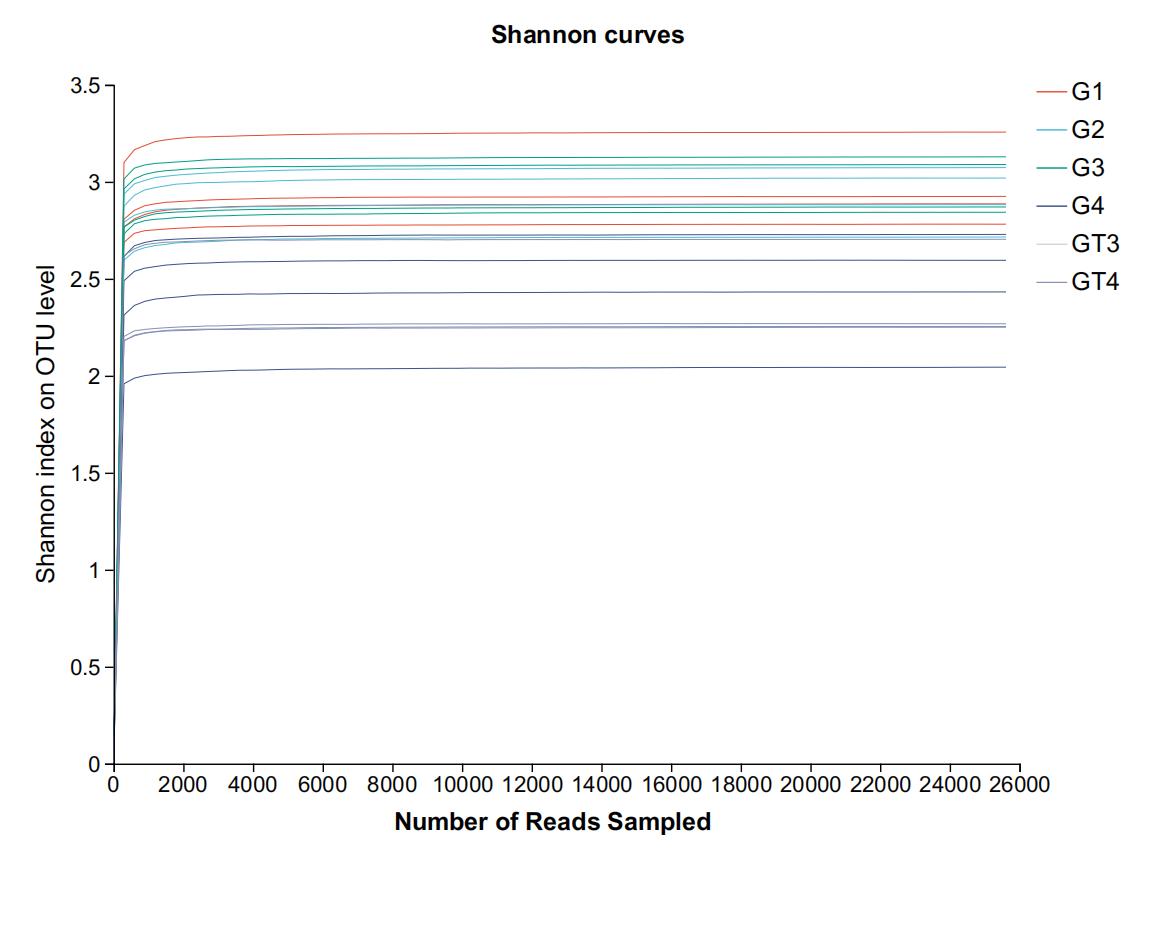

Supplement: Supplementary file 3 [file Image_2.jpeg]

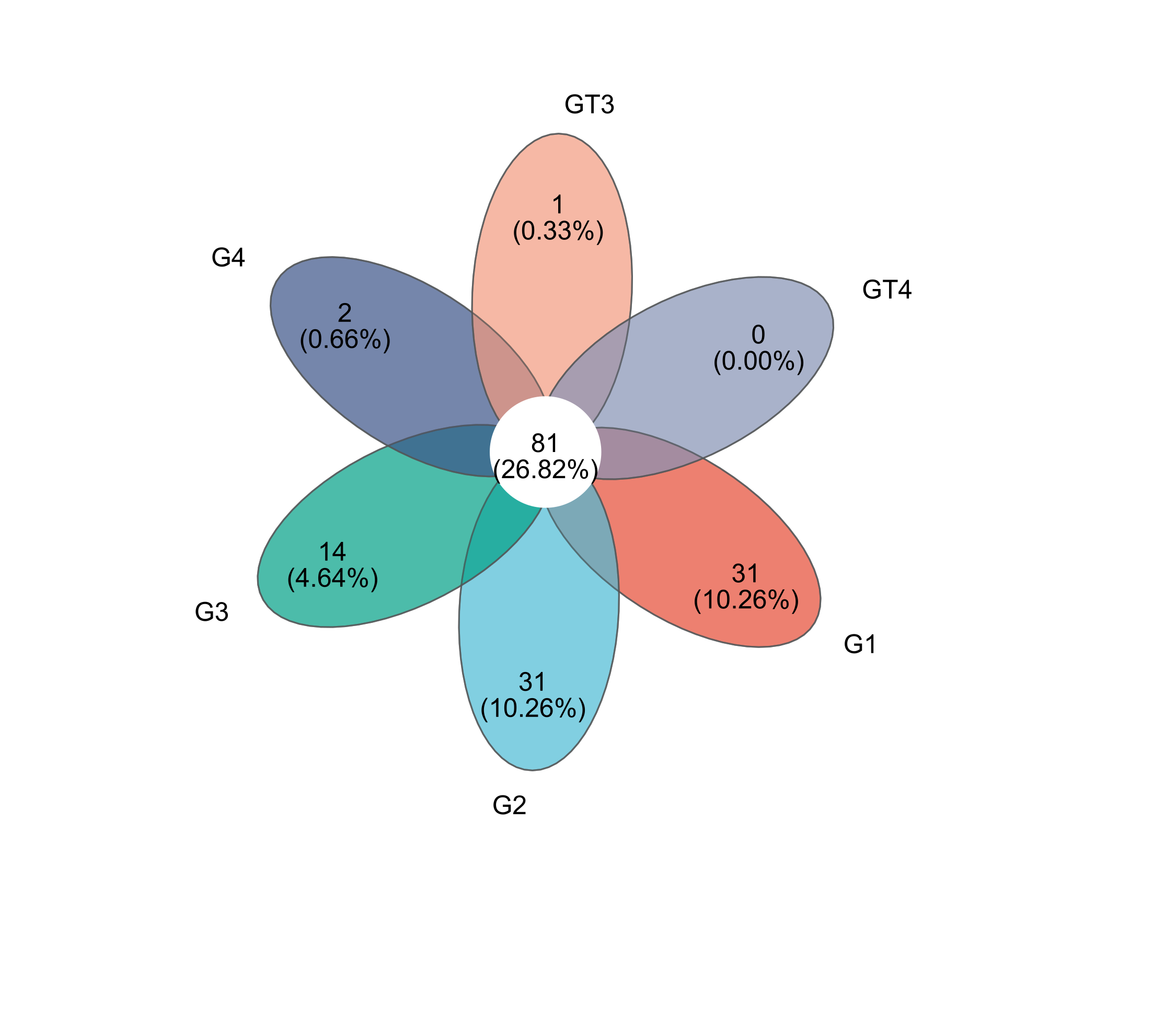

Supplement: Supplementary file 4 [file Image_3.tiff]

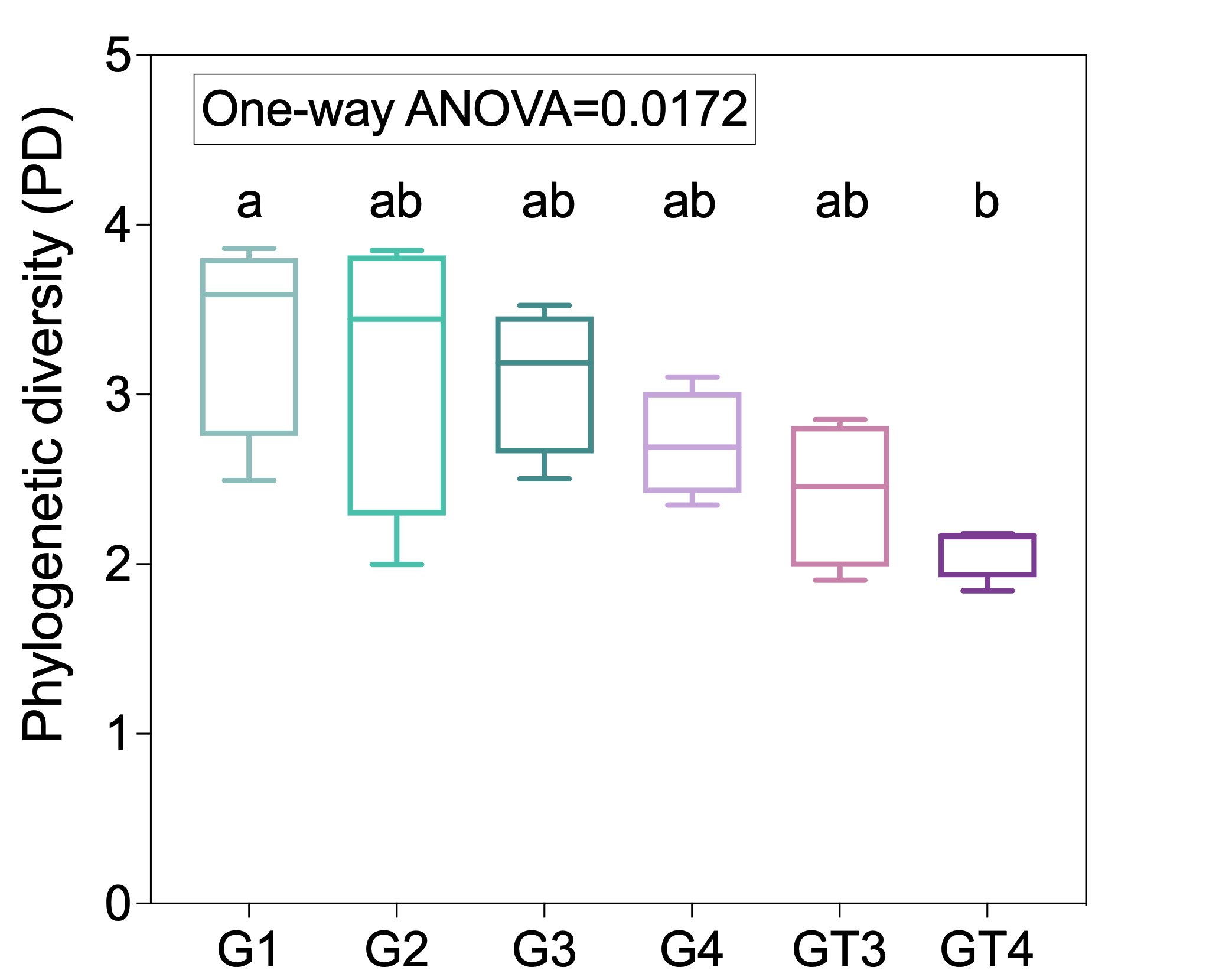

Supplement: Supplementary file 5 [file Image_4.tiff]

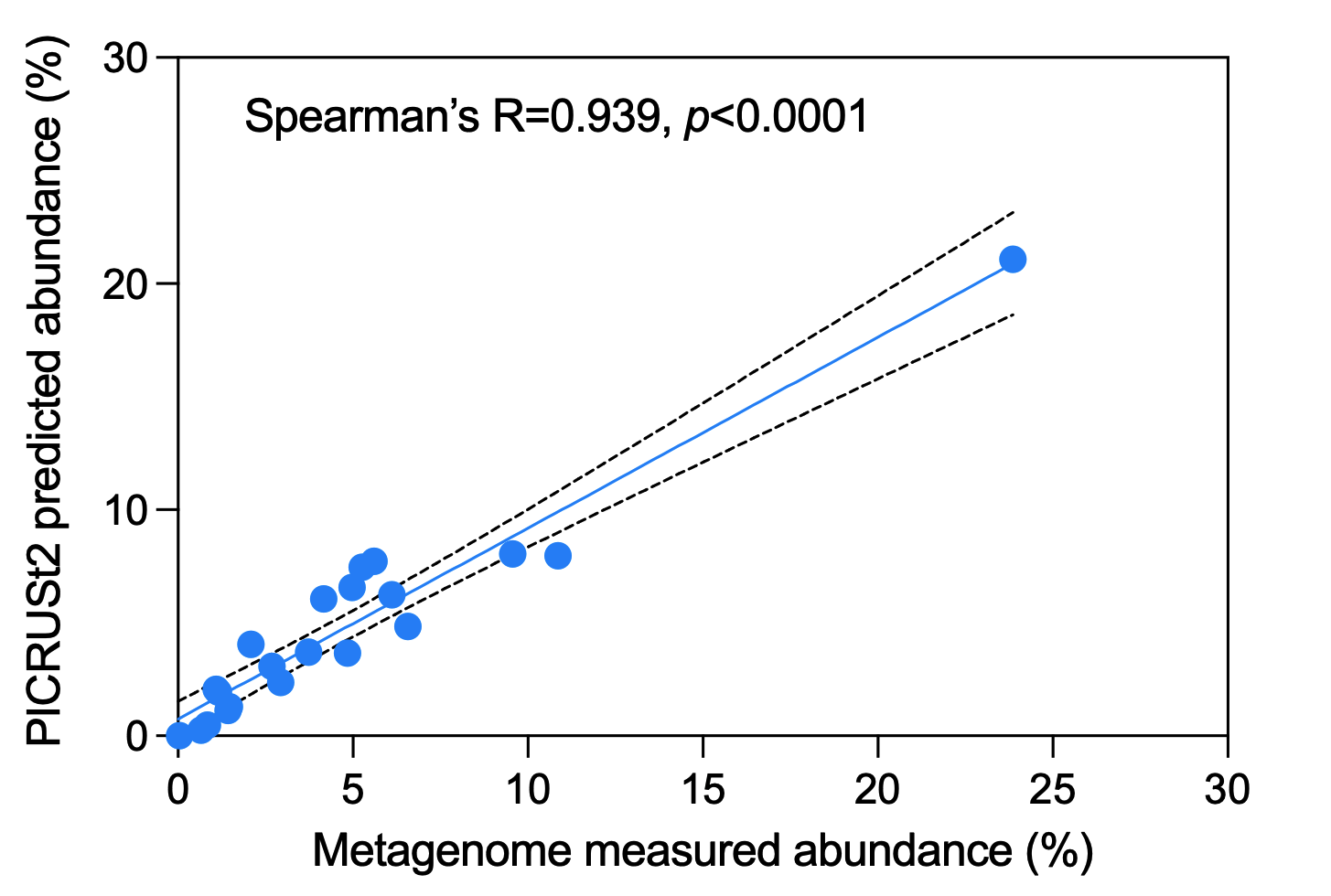

Supplement: Supplementary file 6 [file Image_5.tiff]

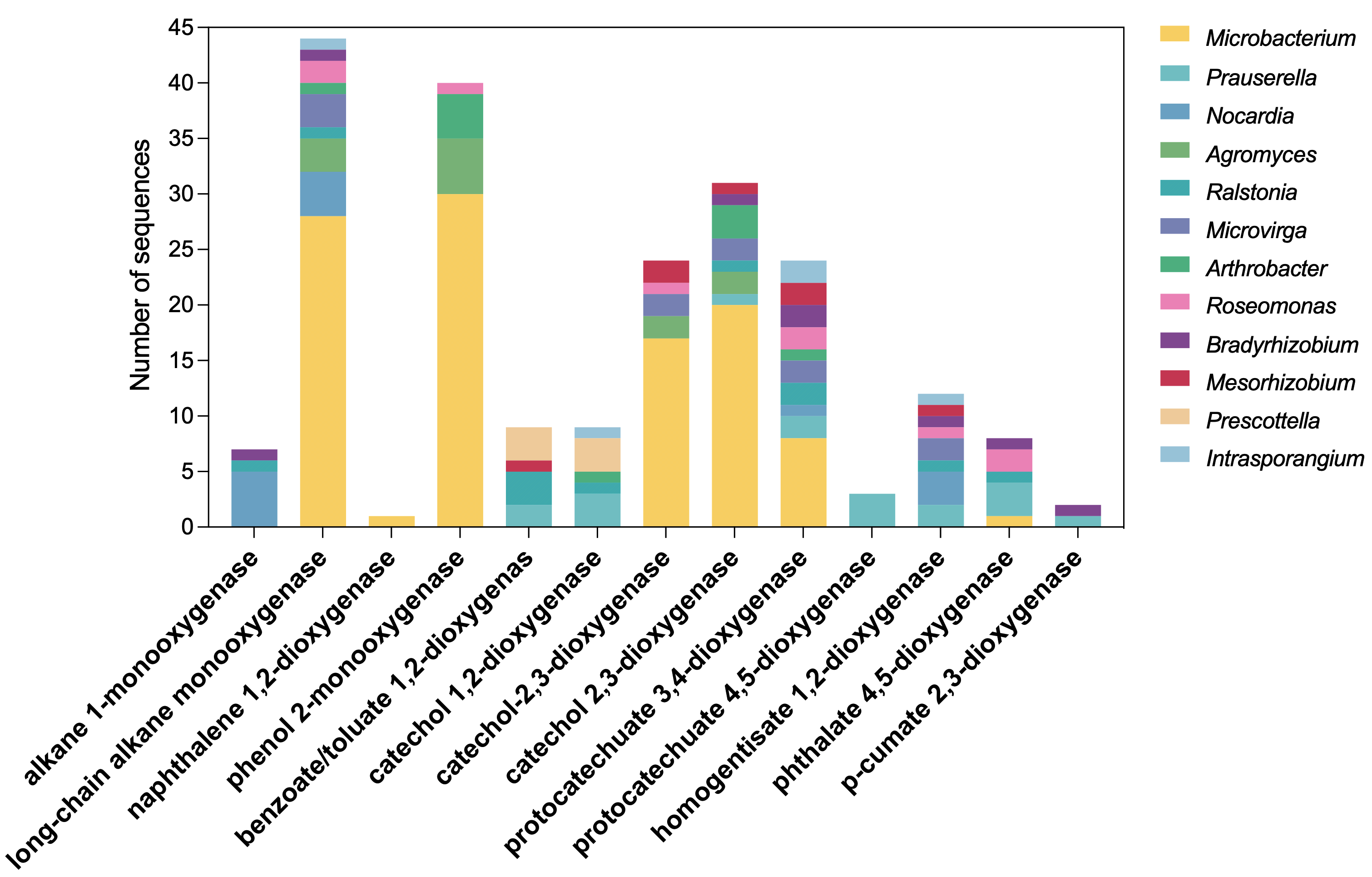

Supplement: Supplementary file 7 [file Image_6.tiff]

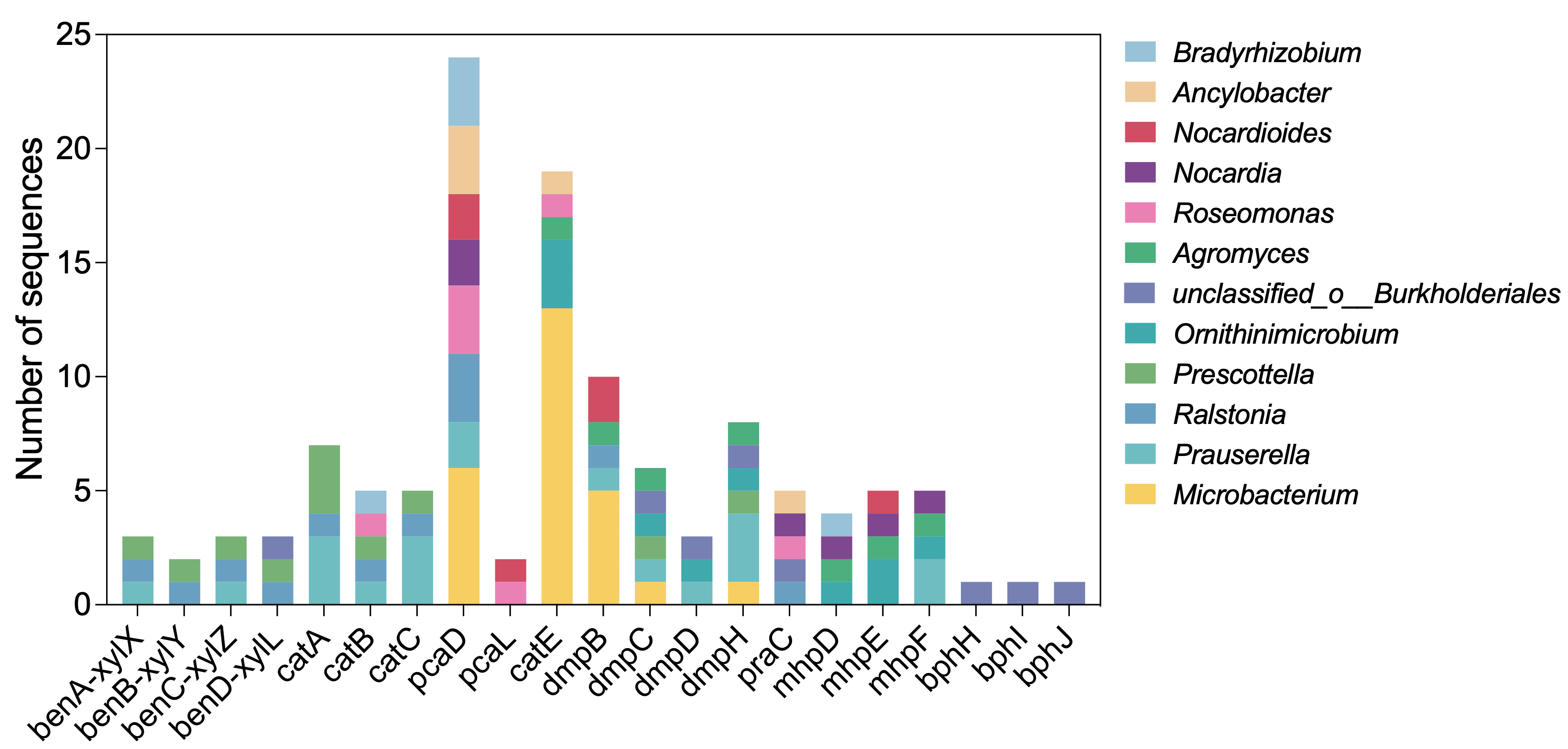

Supplement: Supplementary file 8 [file Image_7.tiff]

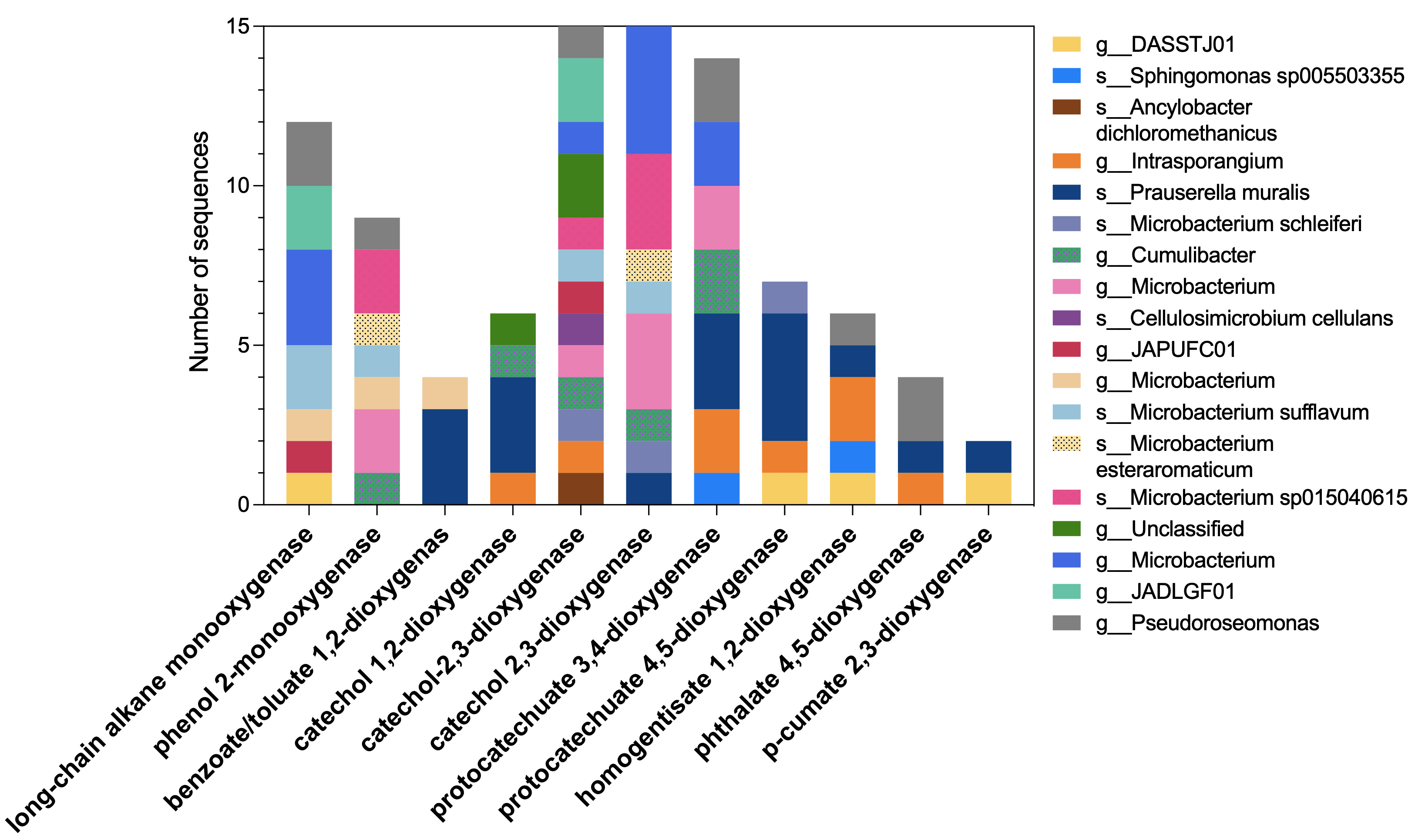

Supplement: Supplementary file 9 [file Image_8.tiff]

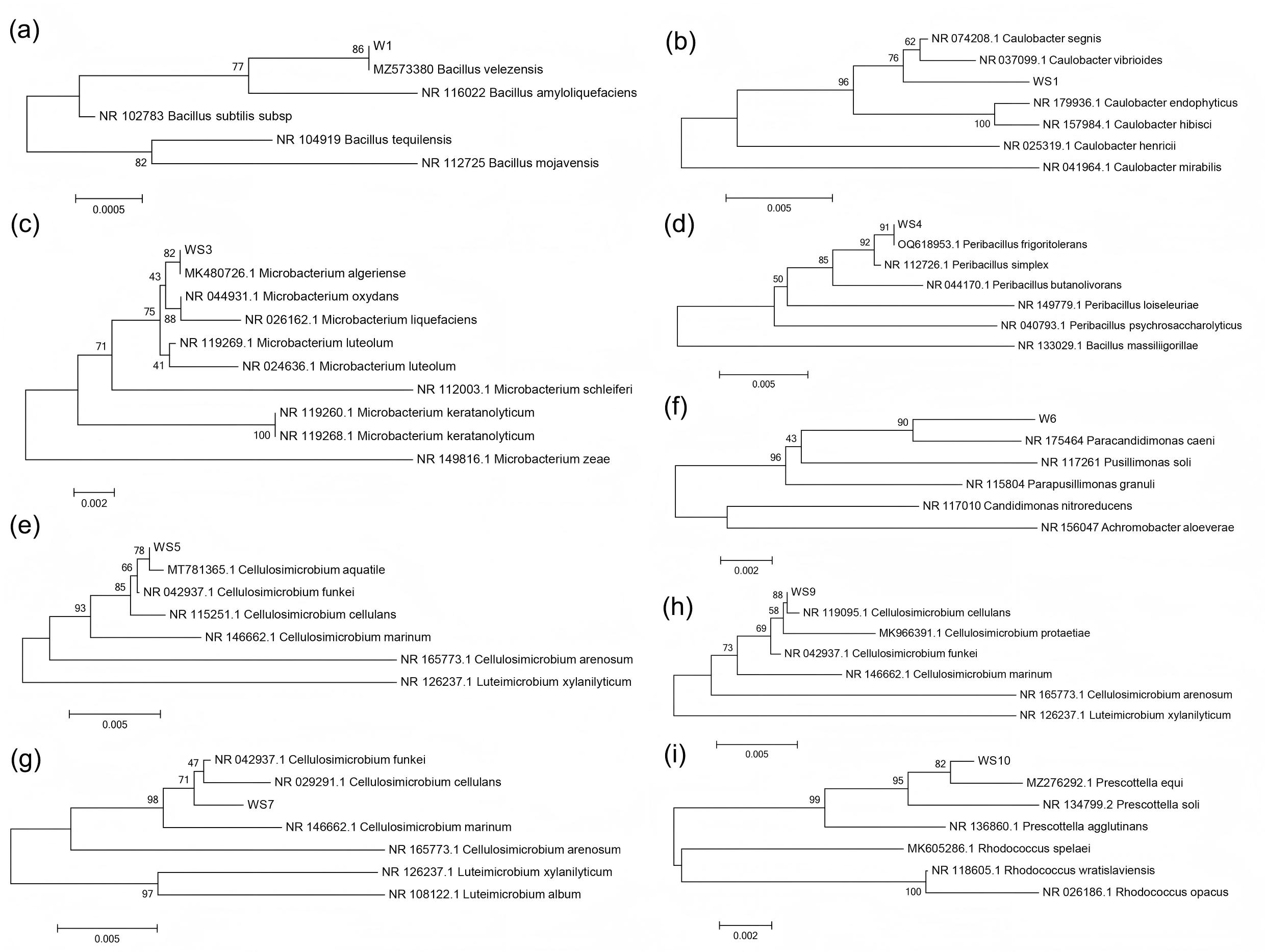

Supplement: Supplementary file 10 [file Image_9.jpeg]

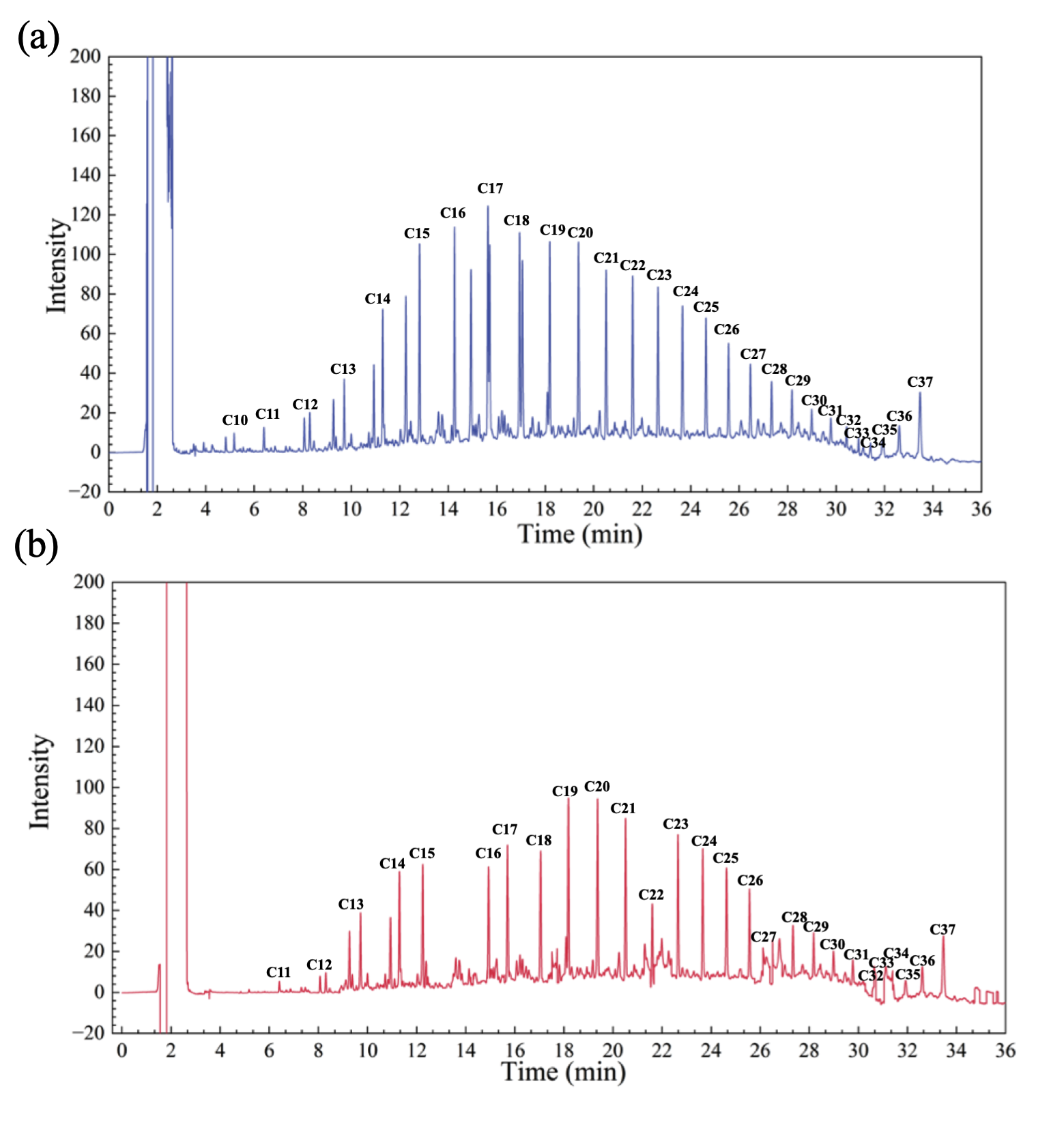

Supplement: Supplementary file 11 [file Image_10.tiff]
